# N-alpha-terminal Acetylation of Histone H4 Regulates Arginine Methylation and Ribosomal DNA Silencing

**DOI:** 10.1371/journal.pgen.1003805

**Published:** 2013-09-19

**Authors:** Vassia Schiza, Diego Molina-Serrano, Dimitris Kyriakou, Antonia Hadjiantoniou, Antonis Kirmizis

**Affiliations:** Department of Biological Sciences, University of Cyprus, Nicosia, Cyprus; Medical Research Council Human Genetics Unit, United Kingdom

## Abstract

Post-translational modifications of histones play a key role in DNA-based processes, like transcription, by modulating chromatin structure. N-terminal acetylation is unique among the numerous histone modifications because it is deposited on the N-alpha amino group of the first residue instead of the side-chain of amino acids. The function of this modification and its interplay with other internal histone marks has not been previously addressed. Here, we identified N-terminal acetylation of H4 (N-acH4) as a novel regulator of arginine methylation and chromatin silencing in *Saccharomyces cerevisiae*. Lack of the H4 N-alpha acetyltransferase (Nat4) activity results specifically in increased deposition of asymmetric dimethylation of histone H4 arginine 3 (H4R3me2a) and in enhanced ribosomal-DNA silencing. Consistent with this, H4 N-terminal acetylation impairs the activity of the Hmt1 methyltransferase towards H4R3 *in vitro*. Furthermore, combinatorial loss of N-acH4 with internal histone acetylation at lysines 5, 8 and 12 has a synergistic induction of H4R3me2a deposition and rDNA silencing that leads to a severe growth defect. This defect is completely rescued by mutating arginine 3 to lysine (H4R3K), suggesting that abnormal deposition of a single histone modification, H4R3me2a, can impact on cell growth. Notably, the cross-talk between N-acH4 and H4R3me2a, which regulates rDNA silencing, is induced under calorie restriction conditions. Collectively, these findings unveil a molecular and biological function for H4 N-terminal acetylation, identify its interplay with internal histone modifications, and provide general mechanistic implications for N-alpha-terminal acetylation, one of the most common protein modifications in eukaryotes.

## Author Summary

The genome of eukaryotic cells is packaged into nucleosomes consisting of an octamer of histone proteins that is wrapped around by DNA. Histone proteins are often modified with chemical groups that can influence the arrangement of nucleosomes and thereby affect DNA-based processes like transcription. Histone N-terminal acetylation, which comprises the addition of a chemical group at the tip of the histone tail, is an abundant modification whose function is unknown. In this work, we show that N-terminal acetylation of histone H4 can strongly inhibit the occurrence of a neighboring modification, namely dimethylation at the third arginine. To do this, N-terminal acetylation cooperates with other internal lysine acetylation marks. We find that the communication amongst these histone modifications is necessary for controlling the expression of ribosomal RNA genes that are required for protein synthesis and cell growth. Our experiments show that in the absence of both N-terminal acetylation and lysine acetylation there is a strong increase in H4 arginine 3 dimethylation levels leading to cell lethality. This growth defect can be rescued by a point mutation on H4 that blocks methylation at position 3. Together, our results unveil a molecular and biological function for the previously uncharacterized N-terminal acetylation of histones.

## Introduction

The nucleosome is the basic unit of chromatin and comprises 147 base pairs of DNA wrapped around a histone octamer, which contains two copies of each of the four histones H2A, H2B, H3 and H4. These histones are subjected to a variety of post-translational modifications, such as methylation, acetylation and phosphorylation, mediated by specific modifying enzymes [1]. Histone modifications often function by recruiting effector molecules to alter the structure of chromatin in order to regulate DNA-based processes such as transcription, replication and DNA repair [1]. An additional property of these modifications is the fact that they cross-talk to each other, whereby one modification influences the establishment or maintenance of a second modification [2].

N-alpha-terminal acetylation is a type of modification occurring on histones. In fact, it is one of the most common protein modifications, present on 80–90% of soluble mammalian proteins and 50–70% of yeast proteins [3], [4]. This mark, which is deposited on the first amino acid residue of the protein has a range of molecular and biological roles, including regulation of protein degradation, protein translocation, protein complex formation, membrane attachment, apoptosis and cellular metabolism [5]. All four core histones [6]–[8] and the linker histone H1 [9] possess N-terminal acetylation, but this modification is more abundant on histones H2A and H4 [8]. N-terminal acetylation of these two histones is mediated by the N-alpha terminal acetyltransferase Nat4 (also known as NatD or Naa40). This enzyme was originally identified in the budding yeast *S. cerevisiae* [7], but also the human ortholog hNaa40 (also designated as hNatD, Nat11 or Patt1) has been recently characterized [10], [11]. Both yeast Nat4 and hNaa40 target only histones H2A and H4, and this specificity differentiates them from all other described N-alpha acetyltransferases, which can target numerous substrates [5].

Previous studies have attempted to determine the biological role of yeast and human Nat4. Deletion of *NAT4* in yeast showed growth sensitivity when cells were cultured in media containing various chemicals such as 3-aminotriazole (3-AT), an inhibitor of transcription [12]. This sensitivity is enhanced when the *NAT4* deletion is combined with mutations in histone H4 where lysines 5, 8 and 12 have been replaced by arginines (K5,8,12R) [12], suggesting that modifications at these residues and N-terminal acetylation are linked through a mechanism that remains elusive. In humans, hNaa40 has been identified as a pro-apoptotic factor and has been implicated in hepatocellular carcinogenesis [11]. Furthermore, a recent study demonstrated that in mice this N-terminal acetyltransferase plays a role in hepatic lipid metabolism [13]. Although some studies have already uncovered phenotypes related to the loss of Nat4 and have provided insights about the biological role of its human ortholog, the molecular function of histone N-terminal acetylation still remains unknown.

Arginine methylation is another histone modification that has attracted much attention in recent years. This is because of its involvement in various cellular processes and the identification of a family of enzymes that catalyze it. These enzymes are called protein arginine methyltransferases (PRMTs) and have already been associated with cancer pathogenesis [14]. PRMTs deposit one or two methyl groups to the guanidino groups of arginine residues resulting in monomethylated (Rme1), asymmetrically dimethylated (Rme2a) or symmetrically dimethylated (Rme2s) states. Others and we have previously shown that arginine methylation cross-talks with adjacent histone modifications by controlling their deposition [15]–[21]. It is, however, also important to discover the mechanisms that regulate PRMT activity and the deposition of histone arginine methylation.

Histone H4 arginine 3 (H4R3) is one of the residues that can possess any of the methylation states [20]. In particular, its asymmetrically dimethylated form is mediated by PRMT1 and it is associated with active transcription in mammals [22], [23]. In yeast, however, the functional homolog of PRMT1 (known as Hmt1) that catalyzes H4R3me2a *in vitro* [24], has been linked to transcriptional repression. Specifically, Hmt1 and its associated H4R3me2a modification have been implicated in the formation of silent chromatin at yeast heterochromatin-like loci, including the rDNA repeat region [25]. This region contains an array of approximately 150 tandem repeats covering approximately 1–2 megabases of chromosome 12 [26]. Within each 9.1 kb rDNA repeat there are two transcriptional units known as *RDN5* and *RDN37* which, respectively, encode for the 5S and 35S rRNAs. The 35S transcript is quickly processed after transcription to generate the 18S, 5.8S and 25S rRNAs [27], [28], which together with 5S are components of a eukaryotic ribosome. Whether the link between H4R3me2a and rDNA silencing relate to the transcriptional levels of these rRNAs is unclear [25].

A previous study has shown that neighboring histone acetylation at lysines 5, 8 and 12 regulates the activity of PRMT1 towards H4R3 *in vitro* [29]. A similar crosstalk among these adjacent modifications has also been proposed to occur in yeast histones [30], but in general the regulation of H4R3me2a *in vivo* remains largely unexplored. Here, we sought to identify factors that control the occurrence of this mark by employing a GPS (Global proteomic screen in *S. cerevisiae*) approach [31]. Using an antibody that specifically detects H4R3me2a we identified Nat4 as an inhibitor of this modification in yeast. Consistent with a role of H4R3me2a in promoting silencing at the rDNA region [25], we find that deletion or inactivation of Nat4 results in enhanced silencing of ribosomal DNA genes. Importantly, we demonstrate that this regulation is mediated through N-terminal acetylation of H4 (N-acH4), but not of H2A. Additionally, we show by using *in vitro* methylation assays that H4 N-terminal acetylation inhibits the activity of the Hmt1 arginine methyltransferase towards H4R3. Interestingly, we find that combinatorial loss of H4 N-terminal and internal K5, 8, and 12 acetylation can induce H4R3me2a deposition even more. Excessive H4R3me2a leads to a severe growth defect, which is rescued by preventing arginine 3 methylation by mutating this residue to lysine. Finally, we provide evidence that the interplay between N-acH4 and H4R3me2a functions under conditions of calorie restriction, which induce rDNA silencing. Altogether, our results reveal the function of H4 N-terminal acetylation in gene regulation, and elucidate the underlying molecular mechanism that links this N-terminal acetylation to other internal histone modifications.

## Results

### Nat4 is a novel regulator of H4R3me2a

**Figure 1:**
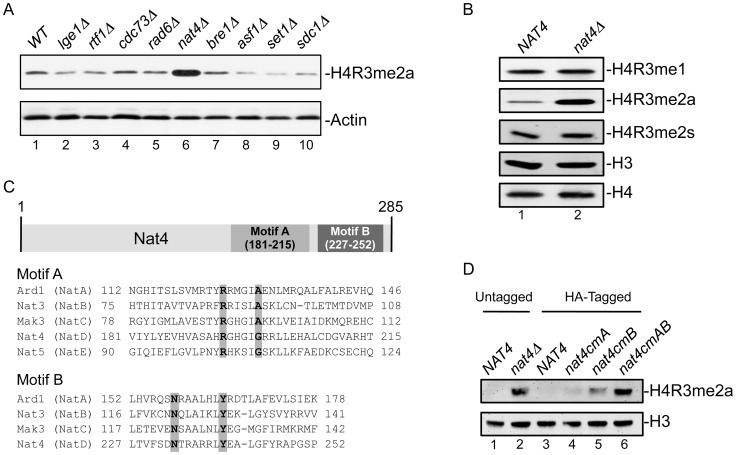
Deletion or inactivation of *NAT4* increases the levels of H4R3me2a. *(A)* Whole cell extracts from the indicated deletion strains were analyzed by western blotting with an antibody against H4R3me2a (top panel). Equal loading was monitored using an antibody against actin (bottom panel). *(B)* Whole cell extracts from a wild-type strain (*NAT4*, lane1) and another one carrying a *NAT4* deletion (*nat4Δ*, lane 2) were analyzed using antibodies against various H4R3 methylation states. Equal loading was monitored with H3 and H4 antibodies. *(C)* Sequence alignment of the catalytic motifs A and B of the five *S. cerevisiae* N-alpha acetyltransferases (NATs). The residues that were mutated within each motif to generate the Nat4 catalytic mutants are highlighted in grey. *(D)* Whole cell extracts from strains containing wild-type *NAT4* (lanes 1 and 3)) and different *NAT4* mutants (lanes 2, 4, 5 and 6) were analyzed by western blotting using antibodies against H4R3me2a (top panel) and H3 (bottom panel). The strain in lane 2 represents a Nat4 deletion strain. All catalytic mutant strains (lanes 4–6) have a C-terminal hemagglutinin (HA) tag. The strain containing the mutations within motif A is designated as *nat4cmA* (lane 4), the one with mutations in motif B is *nat4cmB* (lane 5) and the one with mutations in both motifs is noted as *nat4cmAB* (lane 6). Their equivalent wild-type strain contains only the C-terminal HA-tag (lane 3).

We sought to identify proteins that regulate the deposition of asymmetrically dimethylated arginine 3 on histone H4 (H4R3me2a). To do this we developed an antibody that recognizes specifically methylated H4R3 (Figures S1A and S1B) when it is asymmetrically dimethylated (Figure S1C) and performed a GPS screen using the yeast deletion collection. We found that deletion of the N-alpha acetyltransferase 4 (*nat4Δ*) results in robust induction of the H4R3me2a levels (Figure 1A, lane 6). None of the other four yeast N-terminal acetyltransferases (NatA, NatB, NatC or NatE) showed an effect on H4R3me2a when they were deleted (Figure S2A). This effect was specific to the asymmetrically dimethylated form at H4R3, as specific antibodies (Figure S1C) towards monomethylated (H4R3me1) or symmetrically dimethylated (H4R3me2s) states of this residue detected similar levels for these marks between wild-type and *nat4Δ* strains (Figure 1B, compare lane 1 to 2). To determine whether Nat4 regulation towards H4R3me2a was dependent on its N-terminal acetyltransferase activity we constructed a catalytically inactive version of this enzyme. Mutation of four highly conserved residues (Figure 1C) found within the two motifs of its acetyltransferase domain [7] result in increased signal of H4R3me2a, phenocopying *nat4Δ* (Figure 1D, compare lane 2 and lane 6). Notably, the increase of H4R3me2a in Nat4 deficient cells is not due to epitope preference of the H4R3me2a antibody, as it recognizes equally well H4R3me2a peptides that are either N-terminally acetylated or unacetylated (Figure S1C, compare rows 5 and 6). Together, these findings show that Nat4 regulates the levels of H4R3me2a through its N-terminal acetyltransferase activity.

### Loss of Nat4 activity enhances rDNA silencing and H4R3me2a deposition

**Figure 2:**
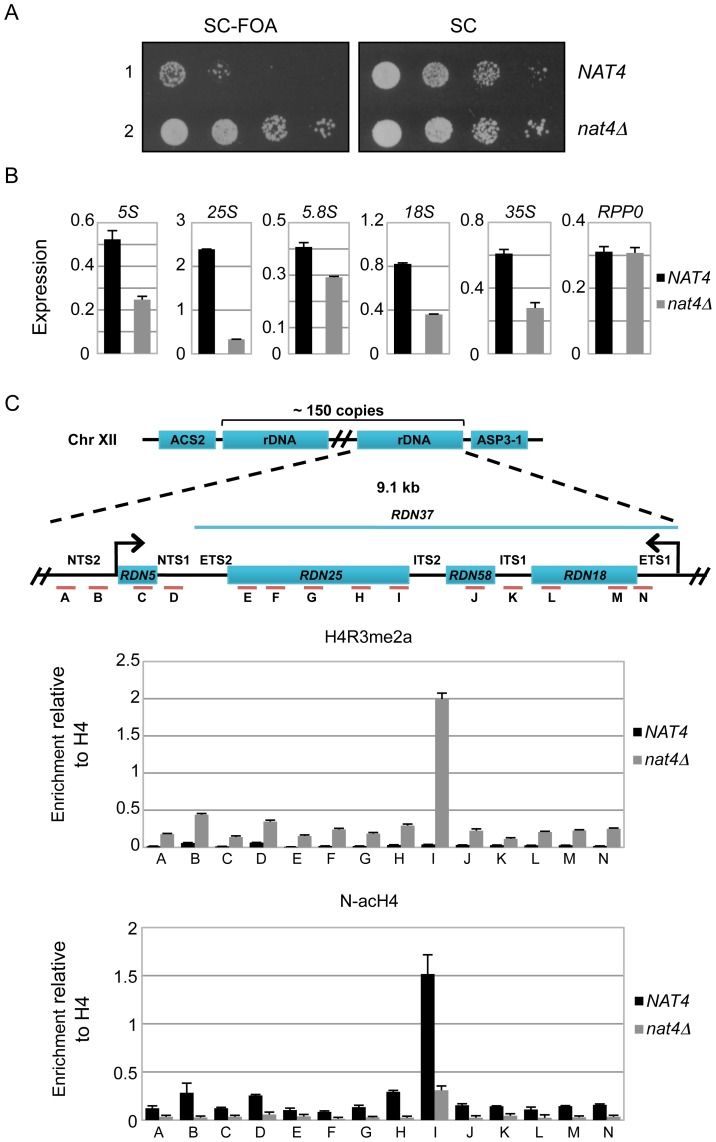
Deletion of *NAT4* enhances silencing and H4R3me2a deposition across the *rDNA* locus. *(A)* Silencing assays for the *rDNA* region were performed with wild-type (row 1) or *nat4Δ* (row 2) strains. Both strains (*NAT4* and *nat4Δ*) carry a copy of the *URA3* gene, that encodes for an essential enzyme in the Uracil metabolic pathway, inserted in the rDNA locus (*RDN1::URA3*). This enzyme metabolizes 5′-Fluoroorotic acid (FOA) into a toxic compound, and the ability of the cell to survive in the presence of FOA depends on the degree of silencing in the rDNA region, such that stronger silencing coincides with more cell growth. The cells were spotted in 10-fold dilutions on SC medium (right panel) or SC+FOA (left panel) and grown for 48 h at 30°C. *(B)* Expression levels of rRNAs 5S, 25S, 5.8S, 18S, 35S and the *RPP0* gene were analyzed by qRT-PCR using total RNA extracted from *NAT4* and *nat4Δ* strains. *(C)* Schematic of the budding yeast rDNA locus on chromosome XII. The rDNA region represents an array consisting of ∼150 tandem copies of a 9.1 kb repeating unit. Each repeat contains the genes *RDN5* and *RDN37* (encodes the 35S primary transcript) as well as two non-transcribed spacers (*NTS1*, *NTS2*), two external transcribed spacers (*ETS1*, *ETS2*) and two internal transcribed spacers (*ITS1*, *ITS2*). Primers were designed along the rDNA locus as indicated by the red lines and letters A–N. ChIP experiments were performed in the *NAT4* and *nat4Δ* strains using antibodies against H4R3me2a (top panel) and N-acH4 (bottom panel). The immunoprecipitated chromatin was analyzed by qRT-PCR using the primers A–N. (see Table S2 for their sequence). The enrichment from each antibody was normalized to the levels of histone H4. Error bars in *(B)* and *(C)* indicate s.e.m for duplicate experiments.

Since a previous study has linked H4R3me2a with the establishment of silencing at the four heterochromatin-like regions in yeast (rDNA, *HML*, *HMR* and telomeres) [25], we sought to determine whether deficiency of Nat4 activity will affect expression at these loci. We found that deletion of *NAT4* did not affect greatly the expression at *HMR*, *HML* and *TEL-VII-L* (Figure S3), but strongly enhances silencing at the rDNA locus (Figure 2A). Due to recent concerns in using FOA-sensitivity assays to assess chromatin silencing [32], [33], we tried to validate the above result by testing the expression of the endogenous rDNA transcripts (Figure 2B). Examining the levels of the different ribosomal RNAs (5S, 25S, 5.8S and 18S, as well as their precursor 35S) by real time-PCR we confirmed the above result, as deletion of *NAT4* significantly reduced the amount of all rRNAs (Figure 2B). It is worth mentioning that because the 35S primary transcript is quickly processed [27], [28], the observed changes in the levels of rRNAs are most likely caused by a decrease in transcription. Interestingly, the deletion of *NAT4* does not affect the mRNA levels of the ribosomal protein Rpp0 (Figure 2B, rightmost panel). A similar result was obtained when the Nat4 catalytic mutant strain (*nat4cmAB-HA*) was used to examine the levels of 25S (Figure S4A), suggesting that rDNA expression is dependent on the Nat4 acetyltransferase activity.

According to these findings, we anticipated that the reduced expression of all rRNAs would correlate with increased deposition of H4R3me2a at the rDNA genes. Indeed, ChIP analysis confirmed that there is higher nucleosomal deposition of H4R3me2a across the entire rDNA locus when Nat4 is absent (Figure 2C, top panel). Consistent with this, an induction of H4R3me2a deposition was also observed at *RDN25* in the strain expressing a catalytically inactivated Nat4 (Figure S4B). As expected, based on the results in figure 1B we did not see changes in the occupancy of H4R3me1 at *RDN25* in the *nat4Δ* strain (Figure S4C). Importantly, the lack of N-terminal acetyltransferase activity in the *nat4Δ* and Nat4 catalytic mutant (*nat4cmAB-HA*) strains was confirmed by an antibody against N-terminally acetylated H4 (N-acH4), which showed that this modification was reduced throughout the rDNA region (Figure 2C, bottom panel and figures S4B–C).

### Nat4 controls rDNA silencing and H4R3me2a through N-alpha-acetylation of H4

**Figure 3:**
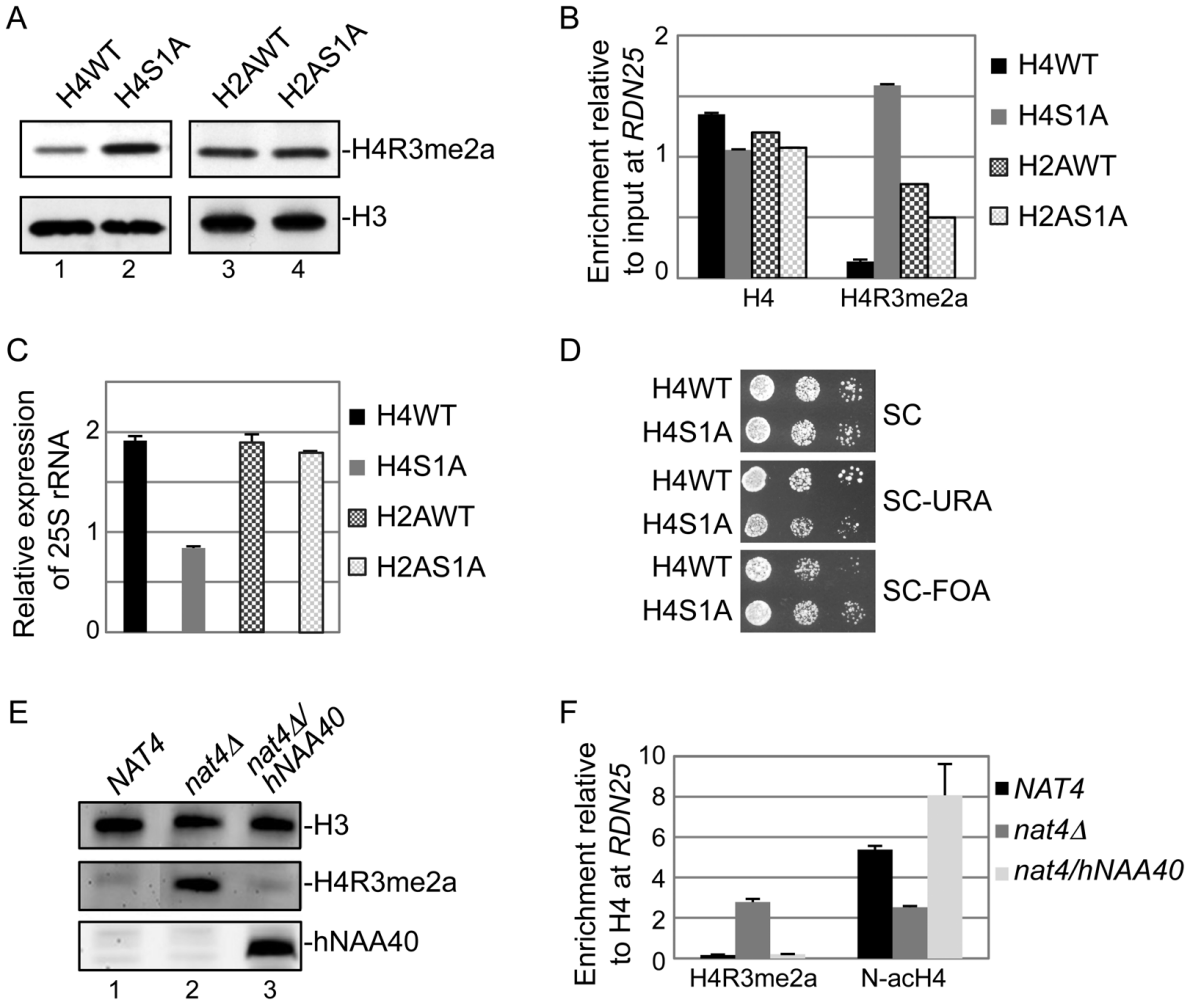
Nat4 inhibits rDNA silencing and H4R3me2a through N-terminal acetylation of H4. *(A)* Whole cell extracts prepared from the wild-type strains (H4WT and H2AWT) and their correspondent Serine-to-Alanine mutants in position 1 (H4S1A and H2AS1A) were analyzed by western blotting using antibodies against H4R3me2a (top panel) and H3 as control (bottom panel). *(B)* ChIP experiments were performed in the same strains as in *(A)* using the H4 and H4R3me2a antibodies. The immunoprecipitated chromatin was analyzed by qRT-PCR using primer I specific to the *RDN25* gene (shown in figure 2C). The enrichment from each antibody was normalized to 1% of the total input DNA. *(C)* Gene expression analysis of 25S rRNA performed using the same strains as in *(A)*. The expression levels of the 25S rRNA were normalized to the levels of *RPP0*. *(D)* Silencing assays for the *rDNA* locus were performed with a wild-type (H4WT) or a H4S1A mutant strain as described in (*2A*). *(E)* Whole yeast cell extracts prepared from the wild-type strains *NAT4* (lane 1), and the mutant strains *nat4Δ* (lane 2) and *nat4Δ/hNAA40* (that carries a *NAT4* deletion and a plasmid that expresses ectopically *hNAA40*, lane 3) were analyzed by western blotting using the indicated antibodies. Equal loading was monitored by an H3 antibody (top panel). *(F)* ChIP experiments performed in the indicated strains as in *(E)* using antibodies against H4R3me2a and N-acH4. The enrichment of each antibody was normalized to the levels of H4 occupancy. Error bars in *(B)*, *(C)* and *(F)* indicate s.e.m for duplicate experiments.

Although our data above demonstrate that Nat4 suppresses rDNA silencing and prevents H4R3me2a deposition, they do not show which one of its two targets, histone H4 or H2A, is implicated in this regulation. To determine this, we constructed yeast strains in which either H4 or H2A were compromised for N-terminal acetylation. Endogenous H4 or H2A were expressed with an alanine instead of a serine (H4S1A or H2AS1A) at the first residue because for both histones, the sequence of their first 30 amino acids is absolutely required for efficient acetylation by Nat4 [7], [12]. Figure 3A shows that mutation of H4 serine 1 to alanine induces H4R3me2a deposition (compare lanes 1 and 2), but the same mutation in H2A has no effect on this methylation (compare lanes 3 and 4). Furthermore, in the H4S1A mutant strain we detected higher amounts of H4R3me2a deposited at the *RDN25* gene compared to an isogenic wild-type (H4WT) strain (Figure 3B). On the other hand, the H2AS1A mutant did not show significant difference in H4R3me2a levels compared to H2AWT strain (Figure 3B). We also like to note that the results of the H4S1A haploid mutant strain are not affected by the fact that H4S1 is also phosphorylated because this modification is only induced under sporulation conditions in diploid cells [34].

To validate that Nat4 regulation of rDNA silencing is mediated through H4, we then examined the expression of this locus in the H4S1A strain (Figure 3C and 3D). Both, analysis of 25S rRNA expression levels and silencing spot assays demonstrate that the H4S1A mutation enhances repression of this locus similarly to *nat4Δ* (compare Figure 3C and 3D to Figure 2B and 2A, respectively), albeit to a lesser extent. This result is in agreement with the increased H4R3me2a levels shown above (Figure 3B). In contrast, H2AS1A mutation does not alter the levels of 25S rRNA (Figure 3C). Additional evidence that rDNA silencing is mediated through N-terminal acetylation of H4 comes from using a strain expressing a H4S1P mutant. The presence of proline at position 1 blocks N-terminal acetylation completely as shown by mass-spectrometry analysis of proteins extracted from yeast, *Drosophila melanogaster* and human cells [3], [35]. As expected, we observed a significant decrease in the levels of 25S rRNA in the H4S1P mutant strain, similarly to the effect observed in the *nat4Δ* strain (Figure S5). Altogether, these results suggest that Nat4 regulates rDNA silencing and H4R3me2a deposition via H4 N-terminal acetylation, but not through N-acH2A.

Finally, to verify the link of N-acH4 within this mechanism, we have also monitored the levels of H4R3me2a in a *nat4Δ* strain that expresses ectopically the human ortholog of Nat4 (hNaa40). It was previously shown that expression of hNaa40 in yeast results in N-terminal acetylation of H4 but not of H2A [10]. In agreement with our data above, we found by western blotting that expression of hNaa40 in a *nat4Δ* strain reduces H4R3me2a back to wild-type levels (Figure 3E, compare lane 3 to lane 1). ChIP analysis also showed that expression of hNaa40 in a *nat4Δ* strain fully restores the N-acH4 levels at *RDN25* (Figure 3F), confirming that histone H4 is the main substrate through which Nat4 regulates H4R3me2a.

### N-acH4 inhibits the Hmt1 methylase activity towards H4R3

**Figure 4:**
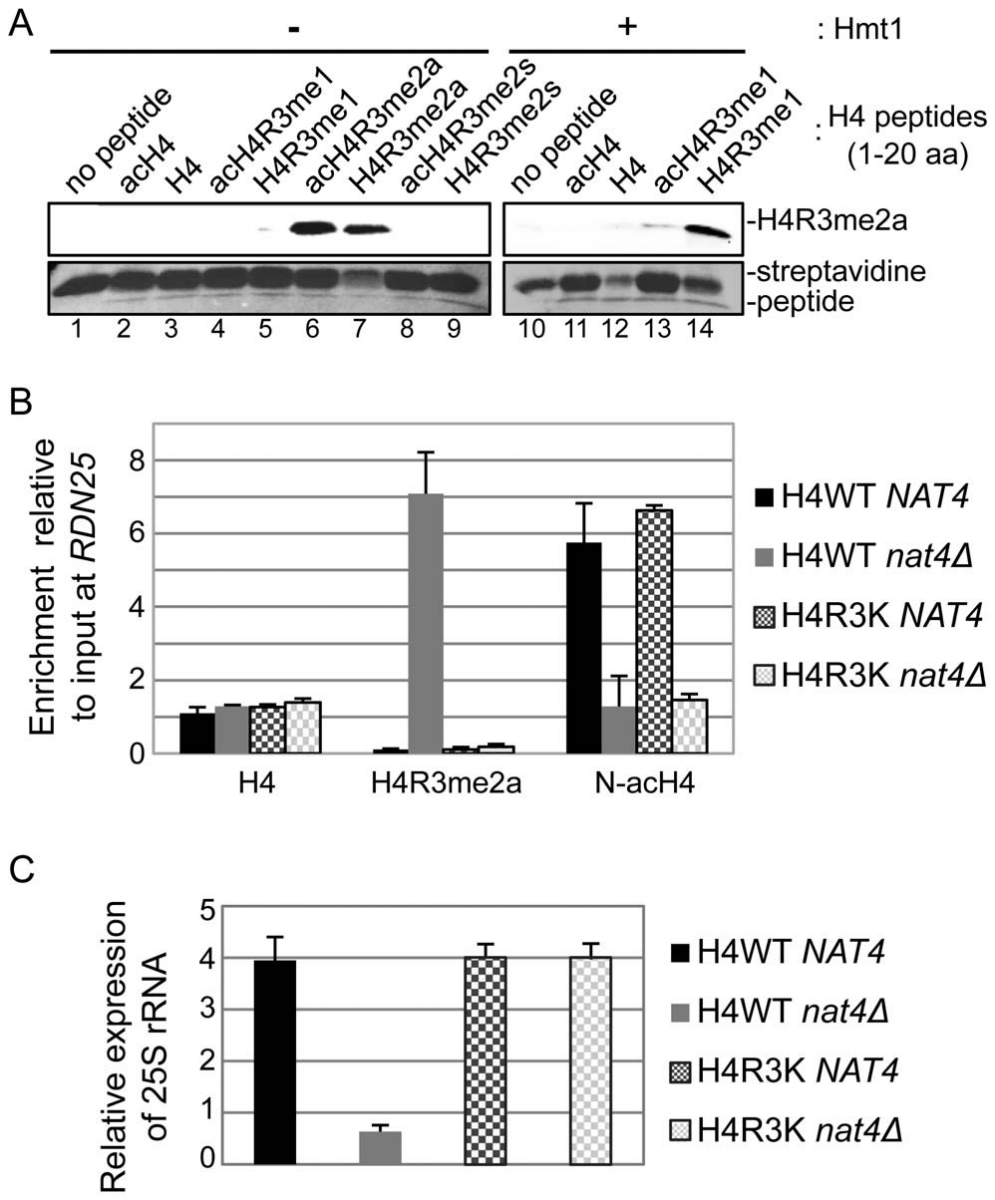
N-acH4 inhibits the Hmt1 methyltransferase activity towards H4R3. *(A) In vitro* methylation assays were performed with synthetic biotinylated peptides representing the first 20 amino acids of histone H4 in the absence (lanes 1 to 9) or presence (lanes 10 to 14) of purified yeast Hmt1. The methyltransferase activity was monitored by western blotting using an antibody against H4R3me2a. Peptide loading was controlled by ponceau staining. *(B)* ChIP experiments were performed in the wild-type (H4WT *NAT4*) and the mutant strains carrying a *NAT4* deletion (H4WT *nat4Δ*), an H4 Arginine-to-Lysine mutation in position 3 (H4R3K *NAT4*) or both (H4R3K *nat4Δ*), using antibodies against H4, H4R3me2a and N-acH4. The enrichment at *RDN25* was analyzed as in (*3B*). *(C)* 25S rRNA expression level analysis was performed in the same strains as in *(B)*. The qRT-PCR analysis was performed as in *(3C)*. Error bars in *(B)* and *(C)* indicate s.e.m for duplicate experiments.

The above results suggest that N-acH4 regulates the deposition of H4R3me2a. To explore this further, we wanted to determine whether the activity of the yeast arginine methyltransferase Hmt1, which was previously shown to target H4R3 *in vitro* [24], is inhibited by N-acH4. We performed methyltransferase assays using Hmt1 purified from yeast cells (Figure S6) and synthetic peptides corresponding to the first twenty amino acids of H4. Immunoblotting for H4R3me2a showed that Hmt1 dimethylates much more efficiently H4R3me1 peptides that are not N-terminally acetylated as opposed to those that possess N-acH4 (Figure 4A, compare lanes 13 and 14). Overall, these findings show that N-acH4 represses the deposition of H4R3me2a by blocking the activity of the associated arginine methyltransferase.

### H4R3 is required for the regulation of rDNA silencing by Nat4 and N-acH4

The previous results link Nat4 with rDNA silencing and H4R3me2a. However, they do not demonstrate whether methylation at H4R3 is necessary and sufficient for the effect of Nat4 towards rDNA expression. To determine this, we investigated the effect of *NAT4* deletion on 25S rRNA expression when arginine 3 was mutated to lysine (H4R3K) in order to prevent its methylation (Figure S1A). Despite the loss of N-acH4 in a H4R3K *nat4Δ* double mutant strain (Figure 4B), the expression levels of 25S rRNA are not reduced compared to the *nat4Δ* only strain (Figure 4C). ChIP analysis at *RDN25* confirms that H4R3me2a is induced in the *nat4Δ* strain, and is undetected in the H4R3K *nat4Δ* strain (Figure 4B). This finding indicates that H4R3 and most likely its methylation are absolutely required for the control of rDNA silencing by Nat4 and N-acH4.

### N-acH4 cooperates with H4K5,-K8,-K12 acetylation to control H4R3me2a, rDNA silencing and cell growth

**Figure 5:**
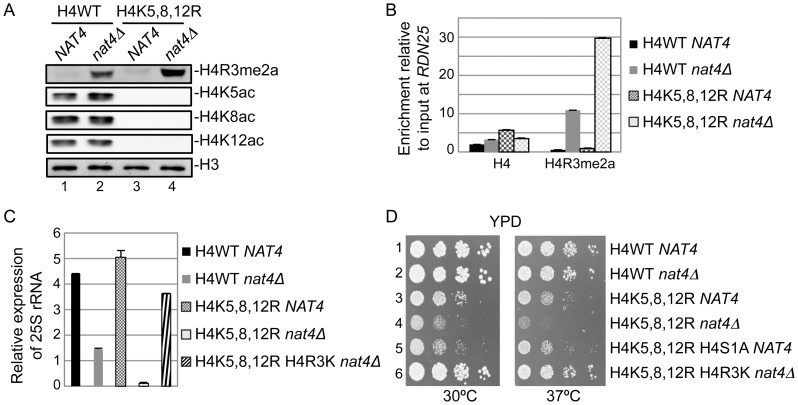
N-acH4 acts synergistically with H4K5, 8, 12 acetylation to control rDNA silencing, H4R3me2a and cell growth. *(A)* Whole yeast cell extracts were prepared from the wild-type (H4WT *NAT4*) and the mutant strains carrying a *NAT4* deletion (H4WT *nat4Δ*), a triple Lysine-to-Arginine mutation in H4 in positions 5, 8 and 12 (H4K5,8,12R *NAT4*) or both (H4K5,8,12R *nat4Δ*) and then analyzed by western blotting using the H4 modification antibodies are shown. Equal loading was monitored with an H3 antibody (bottom panel). *(B)* ChIP experiments were performed in the same strains as in (*A*) using the antibodies against H4 and H4R3me2a. The enrichment of each antibody was analyzed as in *(3B)*. *(C)* 25S rRNA expression level analysis was performed in the wild-type (H4WT *NAT4*) and the mutant strains carrying a *NAT4* deletion (H4WT *nat4Δ*), a triple H4 Lysine-to-Arginine mutation in positions 5, 8 and 12 (H4K5,8,12R *NAT4*) both (H4K5,8,12R *nat4Δ*), and a multiple H4 mutant with a triple Lysine-to-Arginine substitution in positions 5, 8 and 12, an Arginine-to-Lysine mutation in position 3, and a *NAT4* deletion (*H4Κ5,8,12R H4R3K nat4Δ*). The analysis was performed as in (*3C*). Error bars in *(B)* and *(C)* indicate s.e.m for duplicate experiments. *(D)* Growth assay of the same yeast strains as in *(C)* plus a multiple H4 mutant with a triple Lysine-to-Arginine substitution in positions 5, 8 and 12, and Serine-to-Alanine mutation in position 1 (H4K5,8,12R H4S1A *NAT4*). Cells were spotted in 10-fold dilutions on YPAD medium plates. Cell growth was examined at 30°C (left panel) or 37°C (right panel).

Evidence from two previous studies have raised the hypothesis that N-acH4 works together with acetylation of H4K5, H4K8 and H4K12 to control the deposition of H4R3me2a. The first study showed that asymmetric dimethylation of H4R3 mediated by PRMT1 is inhibited *in vitro* by acetylation of lysines 5, 8 and 12 of H4 [29]. The second one demonstrated a synthetic defect in yeast containing *nat4Δ* and a triple lysine to arginine mutant (H4K5,8,12R) [12]. Hence, to explore this hypothesis, we combined *nat4Δ* with the H4K5,8,12R mutant because deletion of Esa1, that acetylates these three lysines is inviable [36]. Interestingly, we found that concurrent loss of N-acH4 and acetylation of H4K5, 8, 12 (H4K5,8,12R *nat4Δ*) results in robust induction of H4R3me2a (Figure 5A, compare lanes 2 and 4), suggesting that these H4 residues collaborate to regulate H4R3me2a. This result is not due to an antibody artifact, as the H4R3me2a antibody recognizes slightly better methylated peptides in which positions 5, 8, and 12 are lysines than when these residues are arginines (Figure S7, compare rows 2 and 3). Because it was recently shown that H4K5, K8 and K12 could also be methylated by Set5 [37], we wanted to investigate the possibility that methylation of these lysines could act synergistically with N-acH4 to control H4R3me2a. Double *nat4Δ set5Δ* deletion did not enhance H4R3me2a levels compared to the *nat4Δ* single mutant (Figure S8, compare lanes 2 and 4), indicating that it is acetylation, and not methylation of H4K5, 8, 12 that cooperates with N-acH4. Notably, N-acH4 is the major regulator of H4R3me2a, as the H4K5,8,12R mutant alone does not increase the levels of H4R3me2a to the same extent as *nat4Δ* (Figure 5A, compare lanes 2 and 3).

To further validate the above results, we also examined the effect of the combination of *nat4Δ* with H4K5,8,12R on the deposition of H4R3me2a and 25S rRNA expression. Consistent with the previous findings we observed a significant enrichment in H4R3me2a at the *RDN25* gene when *NAT4* is deleted together with the H4K5,8,12R mutant as opposed to the *nat4Δ* single mutant (Figure 5B). Moreover, the higher presence of H4R3me2a in the H4K5,8,12R *nat4Δ* double mutant strain results in further reduction of 25S rRNA levels when compared to the *nat4Δ* alone (Figure 5C). Taken together, these results indicate that, both N-acH4 (Figure 4A) and internal lysine acetylation [29] can impede on the methylase activity that targets H4R3, but according to our findings N-acH4 is the predominant regulator of H4R3me2a and rDNA silencing (Figure 5A–C).

Considering that the double mutant of *nat4Δ* with H4K5,8,12R results in robust reduction of 25S rRNA levels (Figure 5C), we then examined the growth rate of this strain using serial dilution spotting assays (Figure 5D) and by measuring its doubling time (Figure S9). Notably, the double mutant strain (H4K5,8,12R *nat4Δ*) has a severe growth defect in comparison to the corresponding single mutants (Figures 5D and S9, left panels). This growth defect becomes lethal when cells are grown at a higher (37°C) temperature (Figures 5D and S9, right panels). Moreover, when H4S1A is combined with the H4K5,8,12R mutant a growth defect is also observed, albeit less severe (Figures 5D and S9, left panels), consistent with the milder deregulation of H4R3me2a and rDNA expression in the H4S1A mutant as opposed to *nat4Δ* (compare Figures 2 and 3). This synthetic defect supports the synergistic effect between N-acH4 and internal H4 lysine acetylation in controlling H4R3me2a and rDNA expression. Based on the previous experiments which showed that arginine 3 is necessary and sufficient for the regulation of rDNA silencing by Nat4 (Figure 4), we then examined whether H4R3K can rescue the growth defect caused by the combination of *nat4Δ* and the H4K5,8,12R mutant. Interestingly, H4R3K rescues entirely the growth defect of the double H4K5,8,12R *nat4Δ* mutant grown at an ambient (30°C) or even at a higher (37°C) temperature (Figures 5D and S9). Additionally, H4R3K restores the rRNA expression levels to almost near wild-type in the double H4K5,8,12R-*nat4Δ* mutant strain (Figure 5C). All together, these results reveal that excessive H4R3 asymmetric dimethylation caused by lack of N-acH4 and internal lysine acetylation impairs cell growth.

### Calorie restriction increases rDNA silencing and the ratio of H4R3me2a to N-acH4

**Figure 6:**
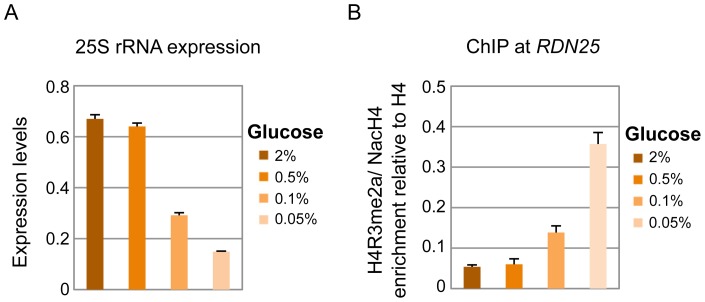
Calorie restriction increases *RDN25* silencing and the H4R3me2a: NacH4 enrichment ratio. *(A)* The levels of 25S rRNA were analyzed by qRT-PCR using total RNA extracted from a wild-type strain (BY4741) grown in minimal media containing different glucose concentrations (2%, 0.5%, 0.1% and 0.05%). *(B)* ChIP experiments were performed in a wild-type BY4741 strain grown in the same conditions as in *(A)*, using antibodies against H4R3me2a and N-acH4. Their enrichment is normalized to histone H4 and represented as ratio of H4R3me2a to N-acH4. Error bars in *(A)* and *(B)* indicate s.e.m for duplicate experiments.

The expression of the rRNA transcripts is modulated by various environmental and intracellular stress conditions. One such condition is calorie restriction, which is studied in yeast by diminishing the levels of glucose in the media. Previous studies have shown that reduction of glucose levels from 2% to 0.5% can enhance rDNA silencing [38], [39]. Hence, we sought to determine whether the crosstalk of N-acH4 and H4R3me2a is induced under these conditions in a wild-type yeast strain. In agreement with previous studies, we found that lowering the glucose availability decreases the levels of 25S rRNA, and this reduction is greater under severe (0.1% and 0.05% glucose) calorie restriction (Figure 6A). Most importantly, the decrease in the amount of 25S rRNA correlates with an increase in the H4R3me2a∶N-acH4 enrichment ratio at the *RDN25* gene. The increase in the enrichment of H4R3me2a against N-acH4 is evident under severe calorie restriction, in line with the lower levels of 25S rRNA (Figure 6B, see 0.1% and 0.05% glucose). These findings suggest that the interplay between H4 N-terminal acetylation and H4R3me2a controls rDNA silencing in response to environmental stimuli such as nutrient deficiency.

## Discussion

**Figure 7:**
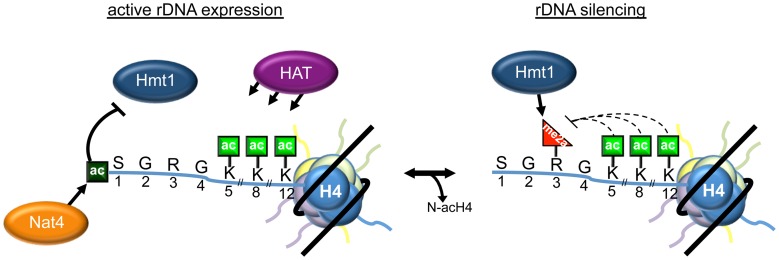
Model depicting the role of N-acH4 in rDNA silencing. When rDNA expression is required, Nat4-catalyzed H4 N-terminal acetylation inhibits Hmt1-mediated H4R3me2a. Under conditions where rDNA expression needs to be repressed, as in an environment with low glucose concentration, N-terminal acetylation decreases by a mechanism still unknown. This mechanism, that can be active (by an enzyme) or passive (by histone dilution) reduces the levels of N-acH4 and allows Hmt1 to asymmetrically dimethylate H4R3, triggering rDNA silencing. Lysine acetylation on residues 5, 8 and 12 fine-tunes the levels of H4R3me2a, as excessive deposition of this mark leads to a severe growth defect at 30°C or even cell lethality at a higher temperature (37°C).

The molecular function of histone H4 N-terminal acetylation was unknown until now, even though this is an abundant and conserved modification that was reported several decades ago [40]. In this study, we describe an important role of N-acH4 in the regulation of histone arginine methylation and rDNA silencing. Taken together, our data support a model in which N-acH4 mediated by Nat4 strongly inhibits the activity of the Hmt1 methyltransferase towards H4R3. This inhibition leads to activation of the rDNA loci. Removal of N-acH4 by a yet unknown mechanism, allows deposition of H4R3me2a and results in repression of rRNA transcription (Figure 7). This mechanism is activated during calorie restriction in order to reduce the expression of the rDNA region in response to the limited source of energy. In the absence of N-acH4, internal lysine acetylation at K5, K8 and K12 catalysed by Esa1 [36] or Hat1 [41] remain unaffected (Figure 5A, compare lanes 1–2 and Figure S10). These acetyl marks can fine-tune the levels of H4R3me2a because otherwise excessive methylation of H4R3 will result in a severe growth defect (Figures 5D and S9). The proposed mechanism also provides an explanation for the previously observed synthetic defect of the double H4K5,8,12R *nat4Δ* mutant strain [12]. Whether the growth defect observed in our experiments is due to deregulation of the rDNA region only or whether other genomic loci whose expression is influenced by H4R3me2a also contribute to this phenotype is still unclear. There are two possible scenarios, which are not mutually exclusive, on how H4R3me2a then mediates rDNA silencing in yeast. First, it was proposed earlier that H4R3me2a facilitates recruitment of Sir2 to the rDNA region [25]. Second, based on previous findings that arginine methylation occludes recruitment of effectors to adjacent modifications [16]–[18], [20], [42], it is possible that H4R3me2a prevents the binding of an activator at the neighboring N-terminal or lysine acetylation marks.

Previous studies suggested that Nat4 and hNaa40 acetylate H4 co-translationally as they were found associated with the ribosomes [10], [12]. However, it remains possible that Nat4 and hNaa40 target H4 post-translationally because a significant amount of hNaa40 localizes to the nucleus [10], [11]. Similarly to other acetyl marks such as H4K5ac and H4K12ac [43], N-acH4 might be catalyzed on soluble nuclear histones that are subsequently incorporated into chromatin. In support of this, N-alpha-terminal acetylation has been proposed to occur post-translationally on other proteins [44], [45]. How N-acH4 is then removed from histones is another pending question. One possibility is through histone exchange by which unacetylated H4 replaces N-terminally acetylated H4 found in chromatin. Another scenario is through active deacetylation mediated by a deacetylase, an activity that has not been demonstrated yet for any protein N-terminal acetylation mark [5].

Interestingly, Nat4 is not the only Nat that has been implicated in the regulation of heterochromatic regions in yeast. NatA has also an active role in chromatin silencing but possibly functions through a mechanism that is distinct from that of Nat4 for three main reasons. Firstly, NatA establishes telomeric and HML silencing by acetylating Orc1 and Sir3 in order to stimulate their recruitment onto chromatin [46]–[48]. However, silencing at the rDNA region does not involve these proteins [49]. Secondly, in our experiments the absence of Ard1 (the catalytic subunit of NatA) has no effect on the levels of H4R3me2a (Figure S2A). Finally, in the *ard1Δ* strain, the levels of 25S rRNA are not significantly altered compared to a wild-type strain (Figure S2B). Therefore, we believe that Nat4 and NatA impact on chromatin silencing through different pathways.

A link between calorie restriction and increased lifespan in yeast and other organisms has already been established [50]. Considering that changes in the levels of N-acH4 and H4R3me2a are associated with calorie restriction (Figure 6), it would be interesting to determine in future studies whether these modifications and their respective enzymes are part of a mechanism that extends cellular lifespan. Another histone H4 acetylation (H4K16ac) has already been implicated in the regulation of lifespan in yeast through a mechanism that maintains telomeric chromatin intact [51]. In contrast, we anticipate that N-acH4 and H4R3me2a, if involved in lifespan regulation, would be part of a pathway that controls rDNA silencing [38], [39], since our data show that deletion of *NAT4* does not affect telomeric silencing (Figure S3). Alternatively, N-acH4, H4R3me2a and their associated enzymes could influence longevity by regulating rDNA recombination, given that Hmt1 activity represses this process [25]. Two recent studies support this idea because they show that calorie restriction suppresses rDNA recombination independently of rDNA silencing in order to extend lifespan [52], [53].

Although this study was performed entirely in yeast, there is evidence suggesting that the cross-talk among N-acH4, internal lysine acetylation and H4R3 methylation may be conserved in mammals. For instance, the activity of Nat4 towards H4 is conserved in humans [10], and its ortholog hNaa40 can re-establish normal levels of H4R3me2a in the absence of Nat4 (Figure 3E–F). Furthermore, mass spectrometry analysis of mouse histone H4 revealed that N-terminal acetylation co-exists with K5, K8 and K12 acetylation and has an inverse relationship with H4R3 methylation [54]. Interestingly, this anticorrelation in mouse cells does not involve asymmetric dimethylation but rather a trimethylated form of H4R3 [54], whose existence is still under debate. The mutual exclusive pattern between N-terminal acetylation and H4R3 methylation becomes even more apparent on H2A peptides [54], suggesting that in mammals this modification crosstalk could also occur on histone H2A. This is consistent with the fact that mammalian H2A (Ser-Gly-Arg-Gly-Lys) has an arginine at position 3 and its N-terminal sequence is identical to H4, in contrast to yeast H2A (Ser-Gly-Gly-Lys-Gly) whose third residue is a glycine. Determining whether Naa40 utilizes a similar mechanism to control gene activation in mammalian cells is intriguing, considering that this enzyme has a pro-apoptotic function and was found significantly downregulated in hepatocellular carcinomas [11].

In summary, this study provides a novel link between protein N-terminal acetylation and the regulation of gene expression. This regulation employs a unique mechanism by which histone N-terminal acetylation influences the deposition of another *in cis* modification. Since N-terminal acetylation occurs on the majority of soluble eukaryotic proteins [3], [4], we propose that its crosstalk with internal post-translational modifications might be a common mechanism for controlling protein function.

## Materials and Methods

### Yeast strains

All strains used in this study are listed in Table S1 and described in Protocol S1.

### Antibodies

Rabbit polyclonal antibodies were raised against H4R3me2a and N-acH4 by Eurogentec (Belgium). Additional details are provided in Protocol S1. Other antibodies used were: H4K5ac (ab51997; Abcam), H4K12ac (ab46983; Abcam), H4K8ac (ab15823; Abcam), H4R3me1 (ab17339; Abcam), H4R3me2s (ab5823; Abcam), H3 (ab1791; Abcam), H4 (62-141-13; Millipore), Naa40 (ab106408; Abcam), b-Actin (ab8226; Abcam) and His-tag (2365; Cell Signalling).

### Growth and silencing assays

Overnight cultures were diluted to OD ∼0.1 and grown to mid-log phase. Approximately 1.2×10^4^ cells were serially diluted 10-fold, and spotted onto the right media plates (YPAD, SC or SC+5′-Fluoroorotic acid). The plates were incubated at 30°C or 37°C for 2 days. Doubling time of cell growth was measured as indicated on http://www.doubling-time.com/compute.php.

### Gene expression analysis

Total RNA from logarithmically grown (OD 0.8) yeast cells was isolated using the hot phenol extraction method [55] and was then treated with the TURBO DNA-free DNase kit (Ambion). Isolated total RNA (0.5 µg) from each sample was mixed with 1 µl dNTP mix (10 mM) and 1 µl of primer cocktail that consists of 0.5 µl oligo-(dT)20 primer (50 µM) and 0.5 µl random hexamers (50 µM) (Invitrogen). DNase RNase-free water was added up to a final volume of 13 µl. The mixture was incubated at 65°C for 5 min for first strand cDNA synthesis. After addition of 4 µl 1× first strand buffer, 1 µl DTT (0.1M), 1 µl RNase inhibitor (RNaseOut 40 U/ µl) and 1 µl Superscript III reverse transcriptase (200 U/µl) (all Invitrogen), the mixture was incubated for 5 min at 25°C, 60 min at 50°C and 15 min at 70°C. A negative control reaction was carried out with 1 µl of DNase RNase-free water instead of the SSIII enzyme. 50 µl of DNase RNAse-free water was added to the final cDNA before analyzing with real-time PCR. SYBR Green (Kapa SYBR Fast Master Mix # KK4602) was used to quantify the level of expression. Relative quantification took place using the reference gene *RPP0* for normalization. Real-time PCR (10 µl reactions) included 1 µl of cDNA, 0.2 µl of forward primer (50 µM), 0.2 µl of reverse primer (50 µM), 5 µl of SYBR Green and 3.6 µl DNase RNase free water. Reactions were incubated in a Biorad CFX96 Real-Time PCR system in 96-well plates using the primers listed in table S2.

### ChIP

ChIP assays were performed as described previously [18].

### Methyltransferase assay

Purified yeast Hmt1 (5 µg) and 22.5 µg of biotinylated histone H4 peptides (Cambridge Peptides, UK) were incubated with 40 µl of MyOne Dynal Streptavidine beads T1 (Invitrogen, #65601) in 100 µl total Reaction Buffer (20 mM Tris-Hcl pH 8, 50 mM NaCl, 1 mM EDTA pH 8, 5% Glycerol, 1 mM DTT, 2 mM S-adenosylmethionine and protease inhibitors) for 20 hours at 30°C with shaking. The beads were then precipitated using a magnetic rack (Invitrogen) and resuspended in 10 µl SDS-loading buffer. The peptides were eluted by alternately boiling, cooling and vortexing the beads three times. The eluted samples were then analyzed by Western blotting and ponceau staining.

### SDS-PAGE and western blotting

Yeast cells were grown to mid-exponential phase in a 30°C shaker. Total yeast extracts were prepared by first resuspending cell pellets in a tenfold volume of SDS loading buffer (50 mM Tris-HCl pH 6.8, 2% SDS, 10% glycerol, 1% β-mercaptoethanol, 12.5 mM EDTA and 0.02% bromophenol blue). The samples were then alternately boiled and chilled three times to rupture cell membranes. Proteins were separated in a 7 cm long, 17% SDS-PAGE (Laemmli 1970) at 200 V for 1 h. The proteins were wet transferred into a PVDF membrane (GE Healthcare life sciences) with 20% Methanol transfer buffer (25 mM Tris, 192 mM glycine, pH 8.3), at 100 V for 1 h. Before incubation with the appropriate antibody, the membrane was blocked in 5% BSA, 0.1% Tween-20 TBS buffer (25 mM Tris, 150 mM NaCl, 2 mM KCl, pH 8).

### Dot blot analysis

Synthesized peptides with at least 90% purity (Cambridge Peptides, UK) were dissolved in water, and drops containing 250, 50, or 10 pmol were deposited on a PVDF membrane, and allowed to air-dry for 1 h. The membrane was then submerged in 100% Methanol for a minute, water for another minute and then stained with Ponceau S or blocked as described above before probing with the appropriate antibody.

## Supporting Information

Figure S1 Specificity of the H4R3me antibodies. *(A)* Whole cell extracts from the indicated wild-type and mutant strains were analyzed by western blotting using an antibody against H4R3me2a. Equal loading was monitored with an H3 antibody. *(B)* Western blot analysis of whole yeast cell extract or recombinant histones H4 and H2A expressed and purified from bacteria. The samples were analyzed with antibodies against H4R3me2a, H4 and H2A. The H4R3me2a antibody recognizes a band in yeast extract that is equivalent to the size of histone H4. *(C)* Dot-blot analysis using synthetic peptides representing the first 20 amino acids of histone H4 and possessing various combinations of R3 methylation and S1 N-alpha-amine acetylation. The peptides were spotted on a PVDF membrane at the indicated concentrations and then probed with antibodies against H4R3me1, H4R3me2a and H4R3me2s. Equal loading of peptides was monitored by Ponceau staining (left panel).
(TIF)

Figure S2 The yeast N-acetyltransferases A, B, C or E do not regulate H4R3me2a. Whole cell extracts prepared from the indicated wild-type and single deletion (*ard1Δ*, *nat3Δ*, *mak3Δ*, *nat4Δ*, *nat5Δ*) strains were analyzed by western blotting using an antibody against H4R3me2a (top panel). The H3 antibody was used as a loading control (bottom panel). *(B)* 25S rRNA expression level analysis was performed with wild-type and the indicated deletion (*nat4Δ* or *ard1Δ*) strains as in *(3C)*. Error bars indicate s.e.m for duplicate experiments.
(TIF)

Figure S3 Deletion of *NAT4* does not affect telomeric, *HMR* or *HML* silencing. Silencing assays were performed as in *(2A)* using *NAT4* and *nat4Δ* strains containing the *URA3* reporter gene integrated at telomere-VIIL, *HMR* or *HML* (*adh4::URA3-TelVII-L*, *hmr::URA3*, or *hml::URA3*). The cells were spotted in 10-fold dilutions on SC medium (right panel) or SC+5′-Fluoroorotic acid (left panel) and then grown for 48 h at 30°C.
(TIF)

Figure S4 The catalytic activity of Nat4 is required to control *RDN25* silencing and H4R3me2a deposition. *(A)* The expression levels of 25S rRNA were analyzed by qRT-PCR as in *(3C)* using total RNA that was extracted from *NAT4-HA* and *nat4cmAB-HA* (for more information about these strains, see *(1C)* and *(1D)*. *(B)* ChIP experiments performed in the strains indicated in *(A)* using H4R3me2a and N-acH4 antibodies and analyzed as in *(3B)*. *(C)* ChIP experiments were performed in *NAT4* and *nat4Δ* strains using antibodies against H4, H4R3me2a, H4R3me1 and N-acH4. The immunoprecipitated chromatin was analyzed as indicated in *(3B)*. Error bars in *(A) (B)* and *(C)* indicate s.e.m for duplicate experiments.
(TIF)

Figure S5 The H4S1P mutant mimics the effect of *nat4Δ*. Gene expression analysis of the 25S rRNA was performed in wild-type (H4WT *NAT4*) and in mutant strains containing a *NAT4* deletion (H4WT *nat4Δ*) or a serine to proline substitution at position 1 of H4 (H4S1P *NAT4*). The expression levels of 25S were normalized to the levels of *RPP0*. Error bars indicate s.e.m for duplicate experiments.
(TIF)

Figure S6 Purification of yeast Hmt1. Immunoblot analysis of purified Hmt1-6His-Ha-ZZ protein using an antibody against the His-tag (right panel). Crude extract (input) prepared from the strain expressing Hmt1-6His-Ha-ZZ was used as a positive control and post-purification extract (depleted) were used to examine the efficiency of the protein purification. Coomassie staining (left panel) was used to monitor protein loading.
(TIF)

Figure S7 The H4K5,8,12R mutation does not enhance recognition by the H4R3me2a antibody. Dot-blot analysis using the indicated synthetic peptides containing the first 20 amino acids of histone H4. The peptides were spotted on a PVDF membrane at the indicated concentrations, and then probed with a H4R3m2a antibody (right panel). Equal loading of peptides was monitored with Ponceau S staining (left panel).
(TIF)

Figure S8 Methylation of H4K5, 8 and 12 by SET5 does not act synergistically with N-acH4 in regulating H4R3me2a. Whole cell extracts prepared from the wild-type (*NAT4 SET5*) and the mutant strains carrying a *NAT4* deletion (*nat4Δ SET5*), a *SET5* deletion (*NAT4 set5Δ*) or both (*nat4Δ set5Δ*) were analyzed by western blotting as in (*S1A*).
(TIF)

Figure S9 The growth defect observed in the H4K5, 8,12R *nat4Δ* strain is rescued by the H4R3K mutation. Cell growth analysis was performed at 30 and 37°C. The strains used are described in *(5D)*. The OD at 600 nm was measured at 0, 1, 2, 4, 6, 8, 10, 20, 24 and 30 h after inoculation of the culture.
(TIF)

Figure S10 Deletion of *NAT4* does not affect the levels of H4K5, 8 or 12 acetylation. ChIP experiments were performed in the indicated strains using antibodies against H4K5ac, H4K8ac and H4K12ac. The immunoprecipitated chromatin was analyzed by quantitative RT-PCR using primers specific to the *RDN25* gene. The enrichment from each antibody was normalized to the occupancy of H4. Errors bars indicate s.e.m for duplicate experiments.
(TIF)

Protocol S1 Additional materials and methods used to construct yeast strains generate antibodies and purify Hmt1.
(DOCX)

Table S1 List of yeast strains used in this study.
(DOCX)

Table S2 List of primer sequences used for qRT-PCR.
(DOCX)
