# Supplementary figures and images for: N-alpha-terminal Acetylation of Histone H4 Regulates Arginine Methylation and Ribosomal DNA Silencing
Source: PLoS Genet. 2013 Sep 19;9(9):e1003805. doi: 10.1371/journal.pgen.1003805 (PMC3778019; doi:10.1371/journal.pgen.1003805)

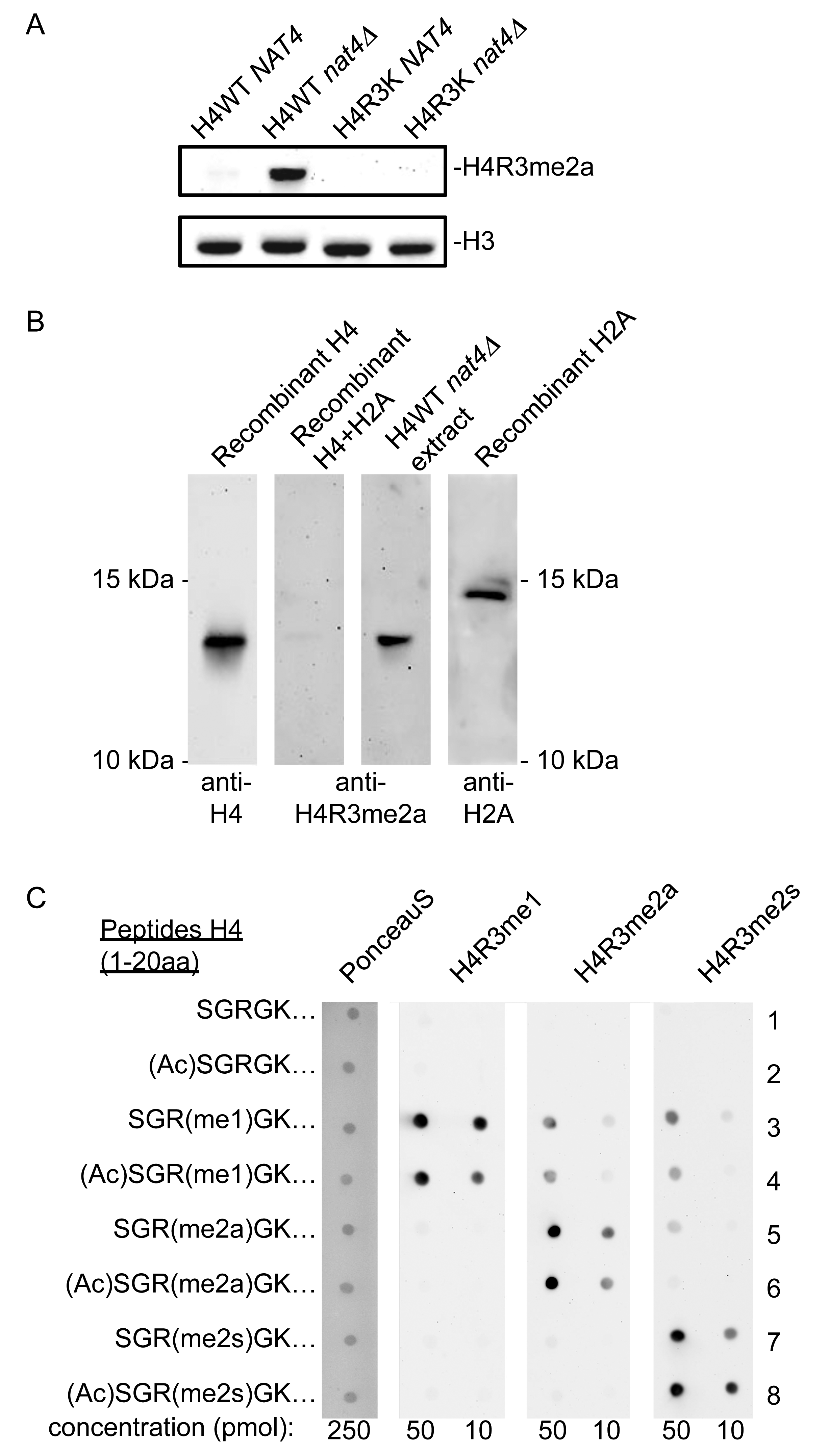

Supplement: Figure S1 — Specificity of the H4R3me antibodies. (A) Whole cell extracts from the indicated wild-type and mutant strains were analyzed by western blotting using an antibody against H4R3me2a. Equal loading was monitored with an H3 antibody. (B) Western blot analysis of whole yeast cell extract or recombinant histones H4 and H2A expressed and purified from bacteria. The samples were analyzed with antibodies against H4R3me2a, H4 and H2A. The H4R3me2a antibody recognizes a band in yeast extract that is equivalent to the size of histone H4. (C) Dot-blot analysis using synthetic peptides representing the first 20 amino acids of histone H4 and possessing various combinations of R3 methylation and S1 N-alpha-amine acetylation. The peptides were spotted on a PVDF membrane at the indicated concentrations and then probed with antibodies against H4R3me1, H4R3me2a and H4R3me2s. Equal loading of peptides was monitored by Ponceau staining (left panel). (TIF) [file pgen.1003805.s001.tif]

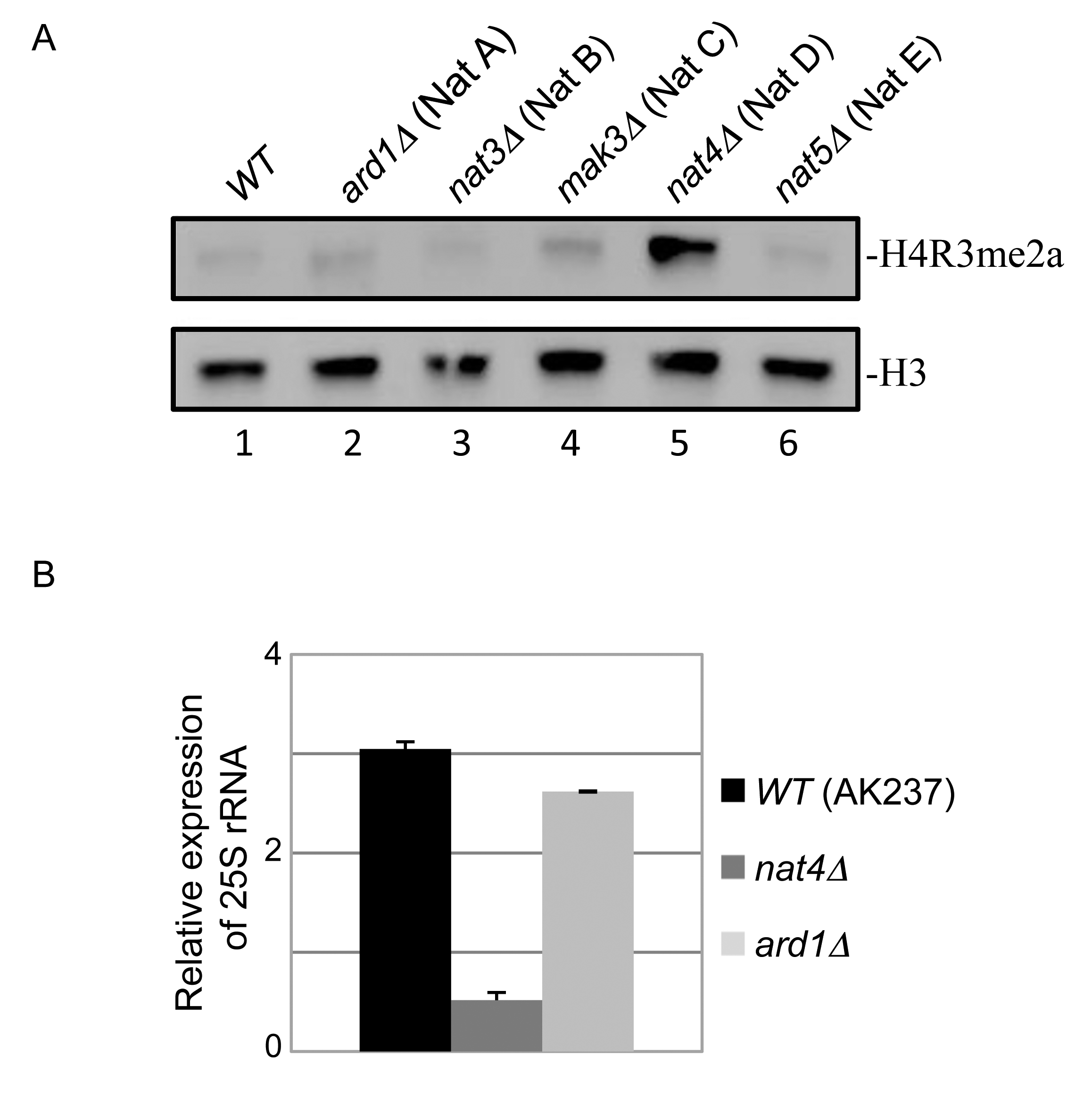

Supplement: Figure S2 — The yeast N-acetyltransferases A, B, C or E do not regulate H4R3me2a. Whole cell extracts prepared from the indicated wild-type and single deletion (ard1Δ, nat3Δ, mak3Δ, nat4Δ, nat5Δ) strains were analyzed by western blotting using an antibody against H4R3me2a (top panel). The H3 antibody was used as a loading control (bottom panel). (B) 25S rRNA expression level analysis was performed with wild-type and the indicated deletion (nat4Δ or ard1Δ) strains as in (3C). Error bars indicate s.e.m for duplicate experiments. (TIF) [file pgen.1003805.s002.tif]

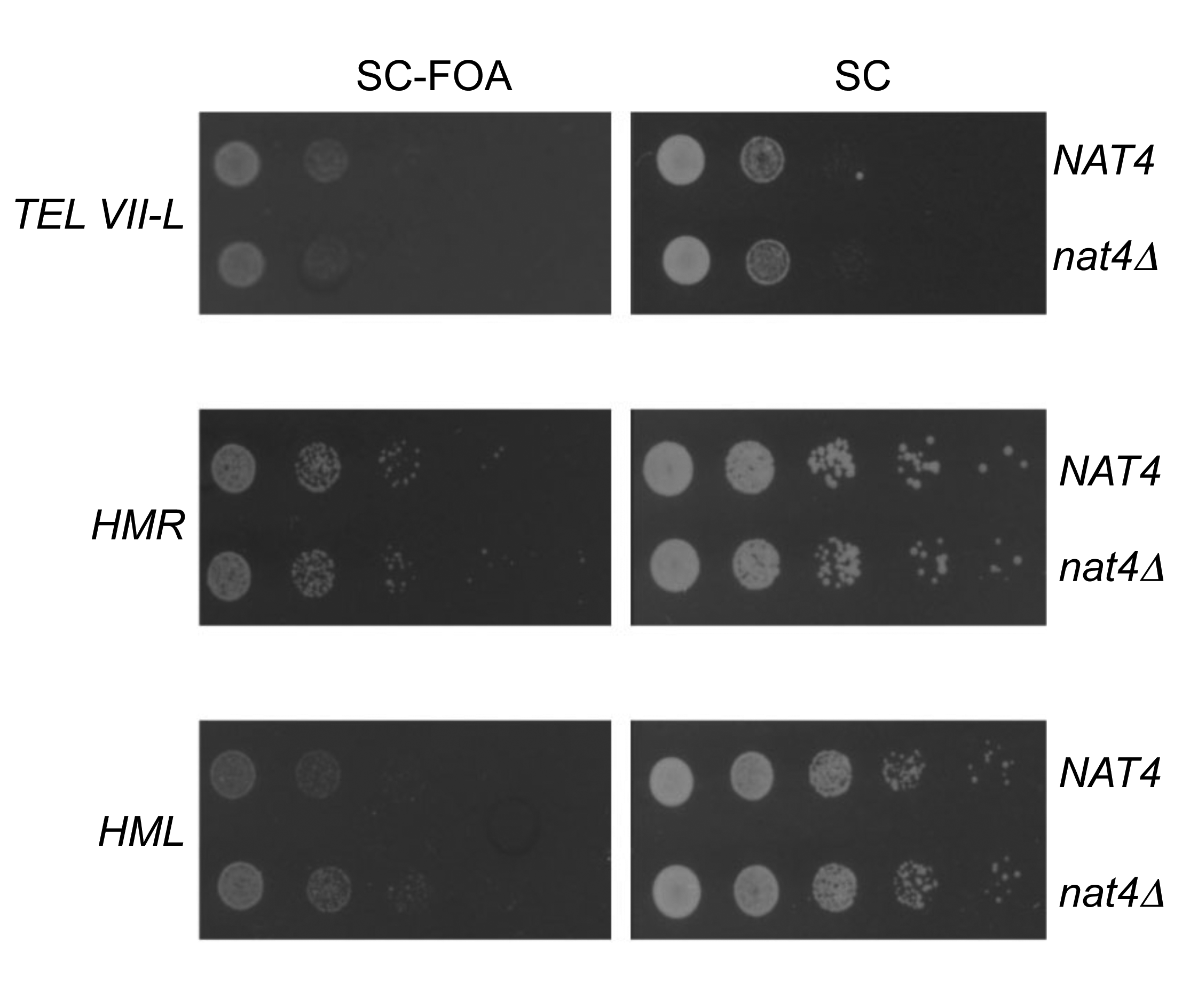

Supplement: Figure S3 — Deletion of NAT4 does not affect telomeric, HMR or HML silencing. Silencing assays were performed as in (2A) using NAT4 and nat4Δ strains containing the URA3 reporter gene integrated at telomere-VIIL, HMR or HML (adh4::URA3-TelVII-L, hmr::URA3, or hml::URA3). The cells were spotted in 10-fold dilutions on SC medium (right panel) or SC+5′-Fluoroorotic acid (left panel) and then grown for 48 h at 30°C. (TIF) [file pgen.1003805.s003.tif]

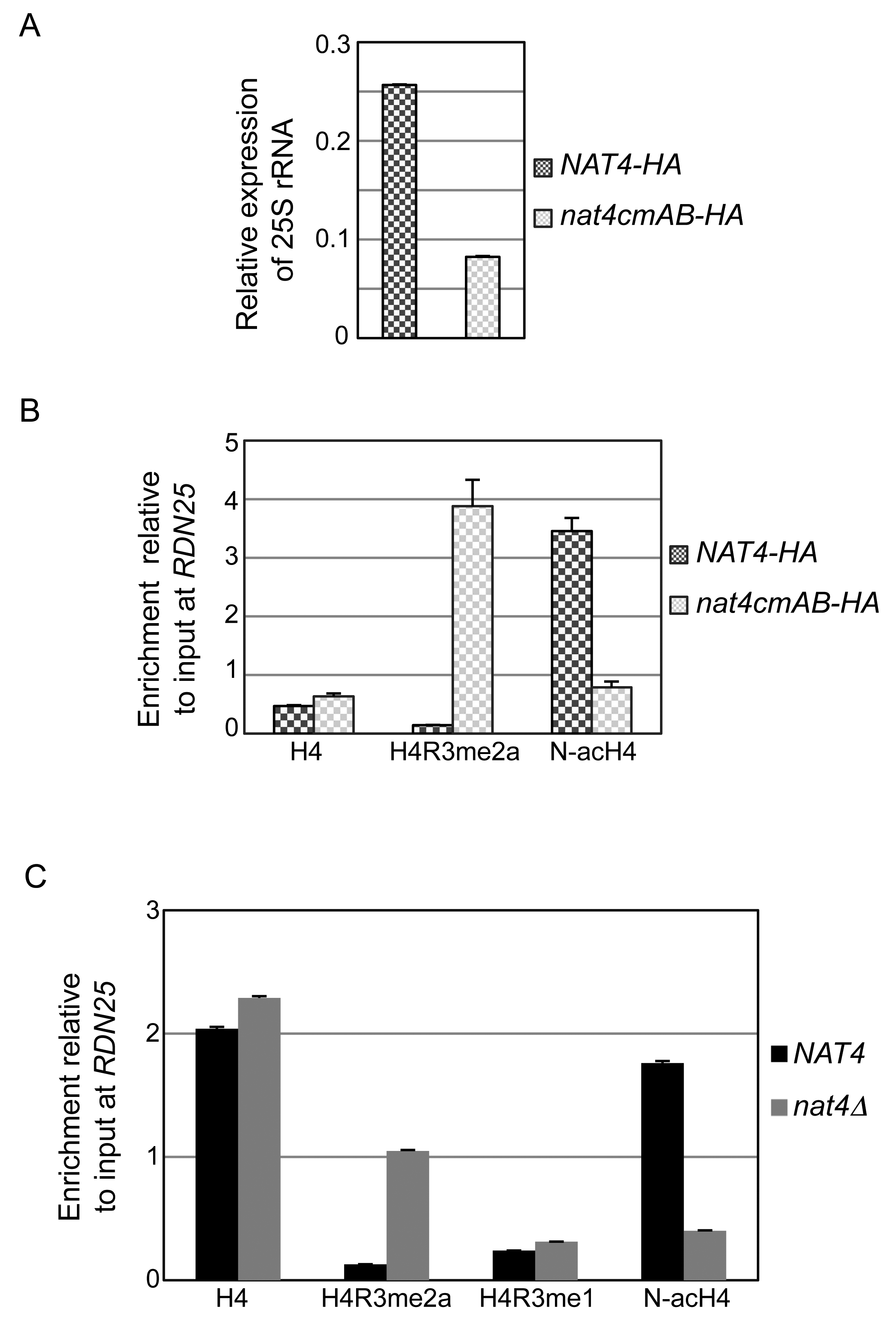

Supplement: Figure S4 — The catalytic activity of Nat4 is required to control RDN25 silencing and H4R3me2a deposition. (A) The expression levels of 25S rRNA were analyzed by qRT-PCR as in (3C) using total RNA that was extracted from NAT4-HA and nat4cmAB-HA (for more information about these strains, see (1C) and (1D). (B) ChIP experiments performed in the strains indicated in (A) using H4R3me2a and N-acH4 antibodies and analyzed as in (3B). (C) ChIP experiments were performed in NAT4 and nat4Δ strains using antibodies against H4, H4R3me2a, H4R3me1 and N-acH4. The immunoprecipitated chromatin was analyzed as indicated in (3B). Error bars in (A) (B) and (C) indicate s.e.m for duplicate experiments. (TIF) [file pgen.1003805.s004.tif]

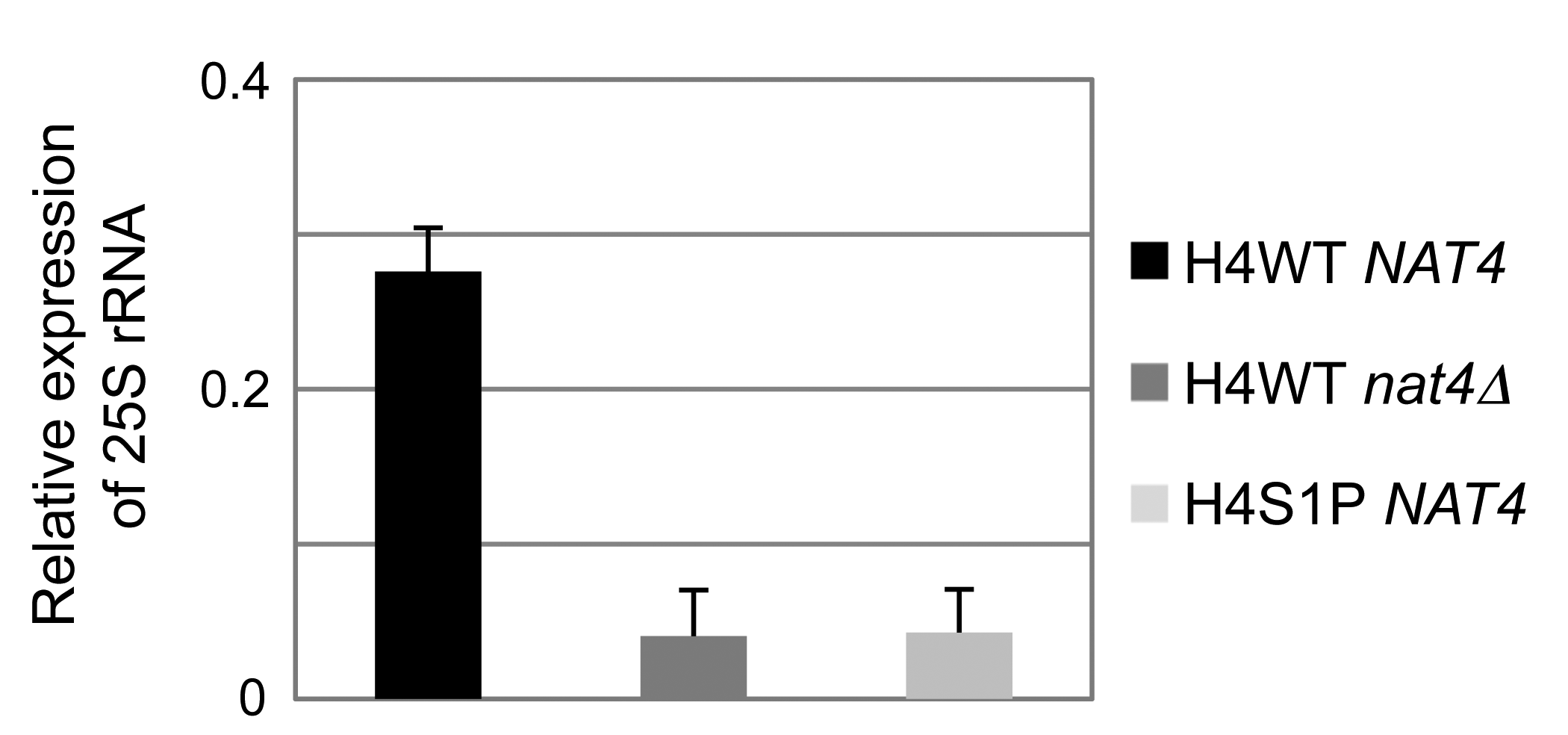

Supplement: Figure S5 — The H4S1P mutant mimics the effect of nat4Δ. Gene expression analysis of the 25S rRNA was performed in wild-type (H4WT NAT4) and in mutant strains containing a NAT4 deletion (H4WT nat4Δ) or a serine to proline substitution at position 1 of H4 (H4S1P NAT4). The expression levels of 25S were normalized to the levels of RPP0. Error bars indicate s.e.m for duplicate experiments. (TIF) [file pgen.1003805.s005.tif]

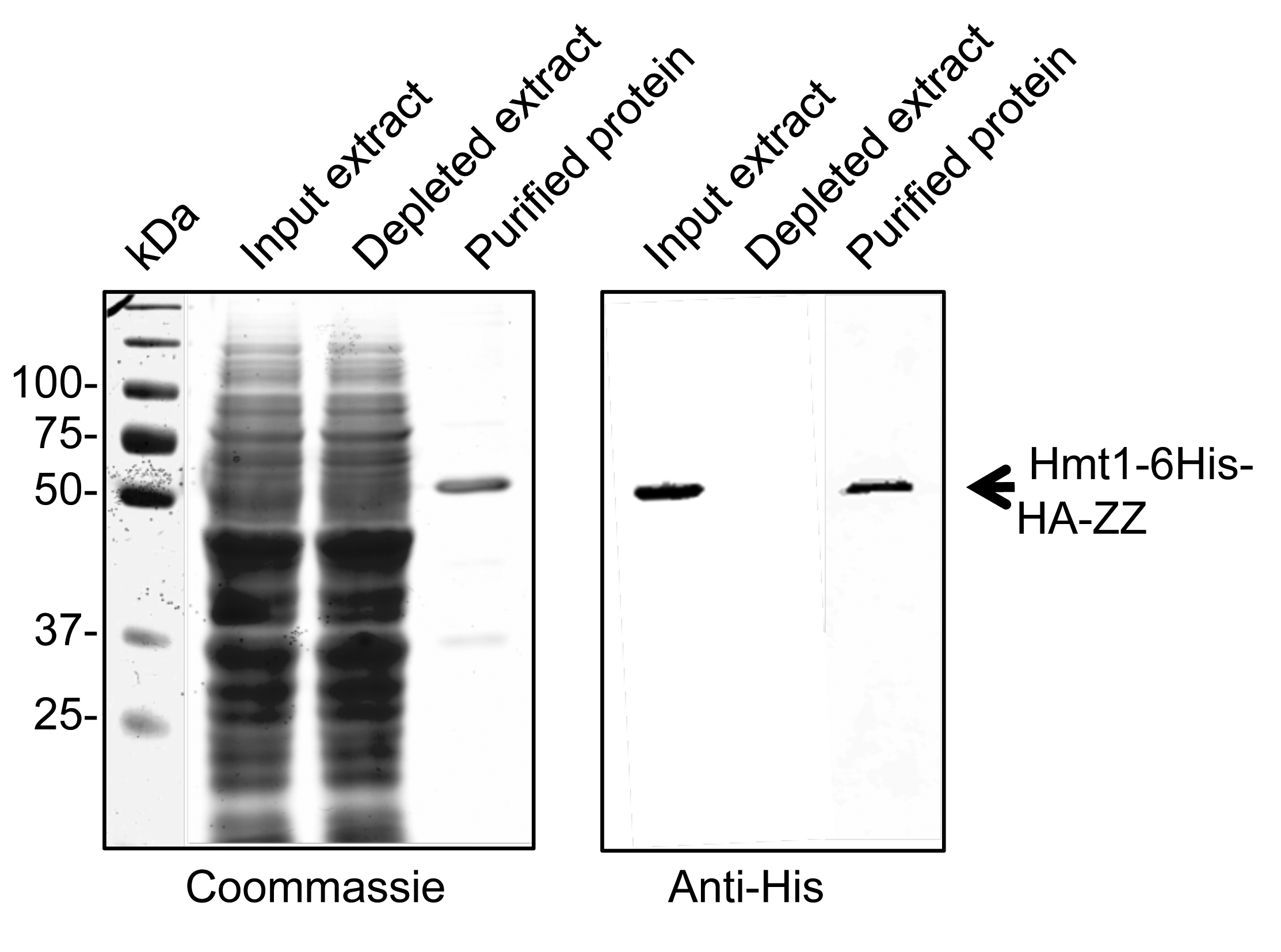

Supplement: Figure S6 — Purification of yeast Hmt1. Immunoblot analysis of purified Hmt1-6His-Ha-ZZ protein using an antibody against the His-tag (right panel). Crude extract (input) prepared from the strain expressing Hmt1-6His-Ha-ZZ was used as a positive control and post-purification extract (depleted) were used to examine the efficiency of the protein purification. Coomassie staining (left panel) was used to monitor protein loading. (TIF) [file pgen.1003805.s006.tif]

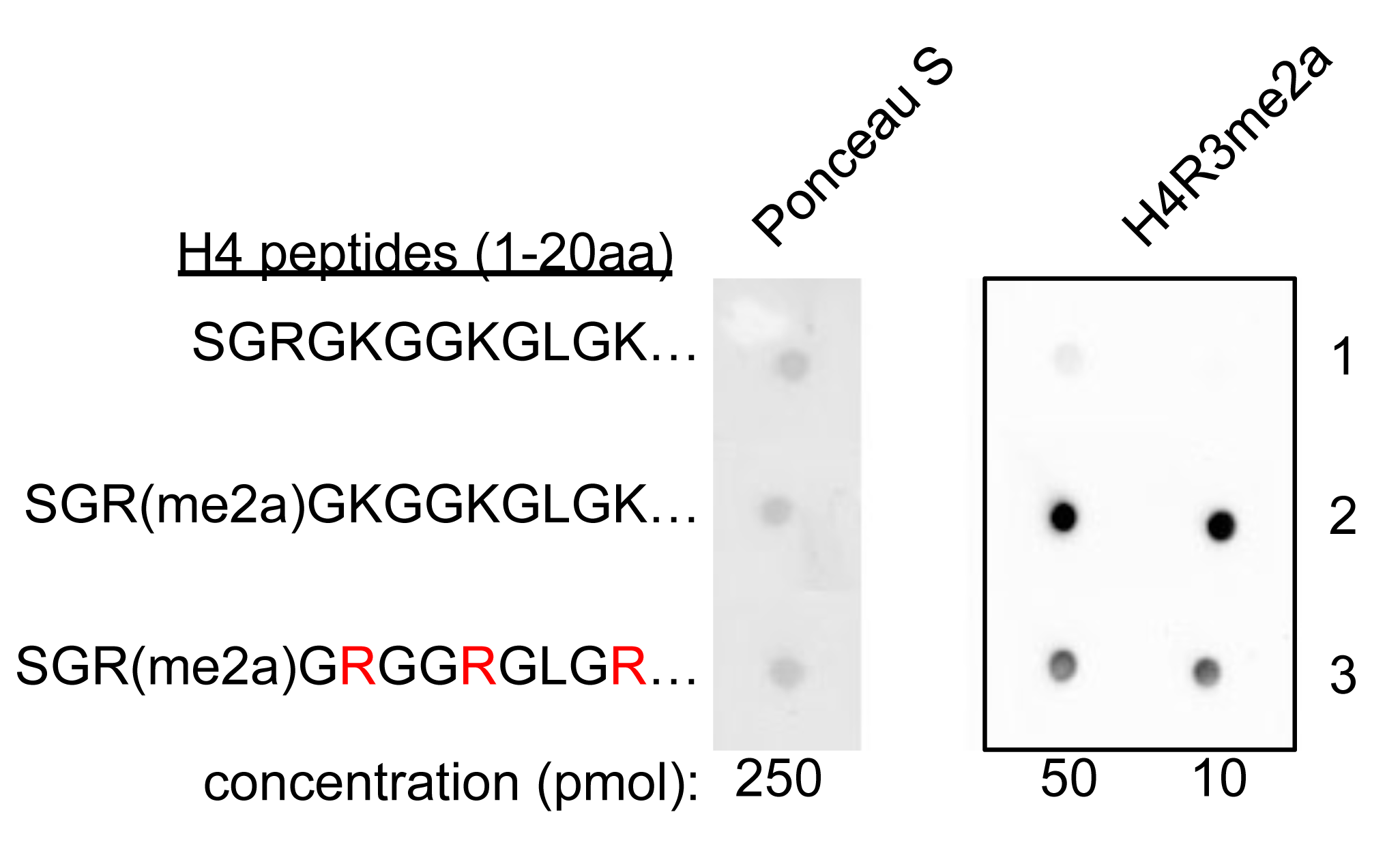

Supplement: Figure S7 — The H4K5,8,12R mutation does not enhance recognition by the H4R3me2a antibody. Dot-blot analysis using the indicated synthetic peptides containing the first 20 amino acids of histone H4. The peptides were spotted on a PVDF membrane at the indicated concentrations, and then probed with a H4R3m2a antibody (right panel). Equal loading of peptides was monitored with Ponceau S staining (left panel). (TIF) [file pgen.1003805.s007.tif]

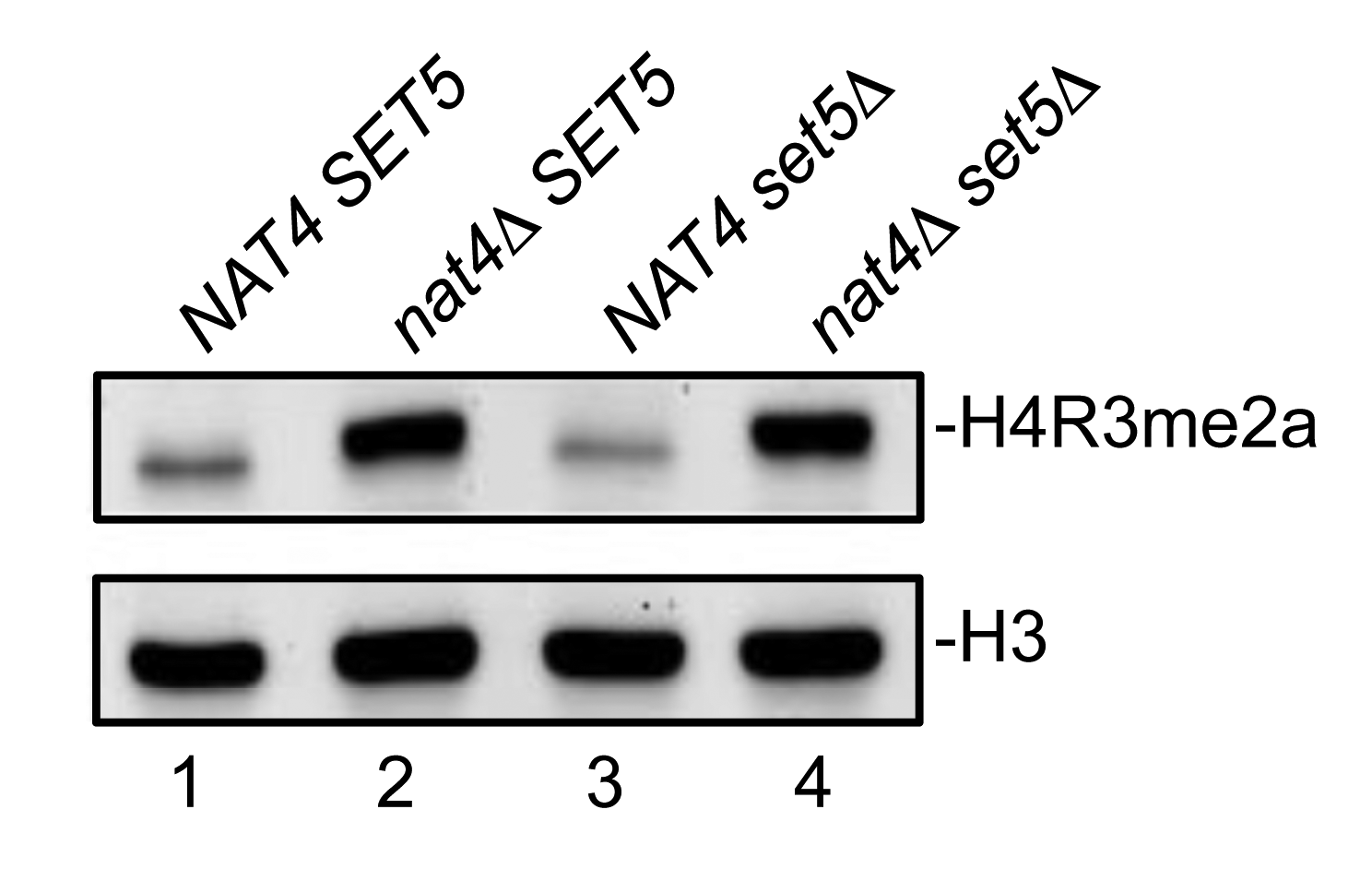

Supplement: Figure S8 — Methylation of H4K5, 8 and 12 by SET5 does not act synergistically with N-acH4 in regulating H4R3me2a. Whole cell extracts prepared from the wild-type (NAT4 SET5) and the mutant strains carrying a NAT4 deletion (nat4Δ SET5), a SET5 deletion (NAT4 set5Δ) or both (nat4Δ set5Δ) were analyzed by western blotting as in (S1A). (TIF) [file pgen.1003805.s008.tif]

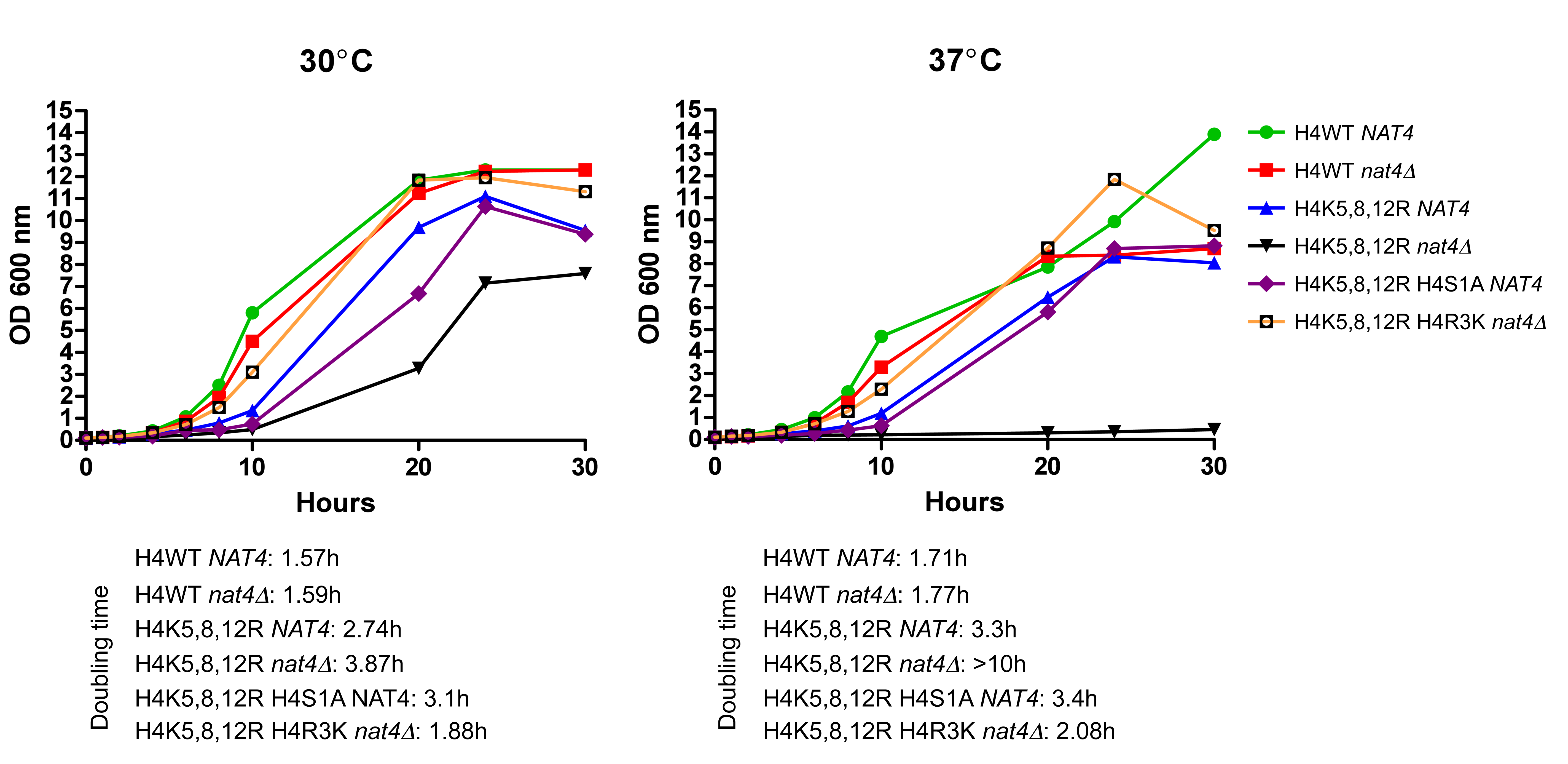

Supplement: Figure S9 — The growth defect observed in the H4K5, 8,12R nat4Δ strain is rescued by the H4R3K mutation. Cell growth analysis was performed at 30 and 37°C. The strains used are described in (5D). The OD at 600 nm was measured at 0, 1, 2, 4, 6, 8, 10, 20, 24 and 30 h after inoculation of the culture. (TIF) [file pgen.1003805.s009.tif]

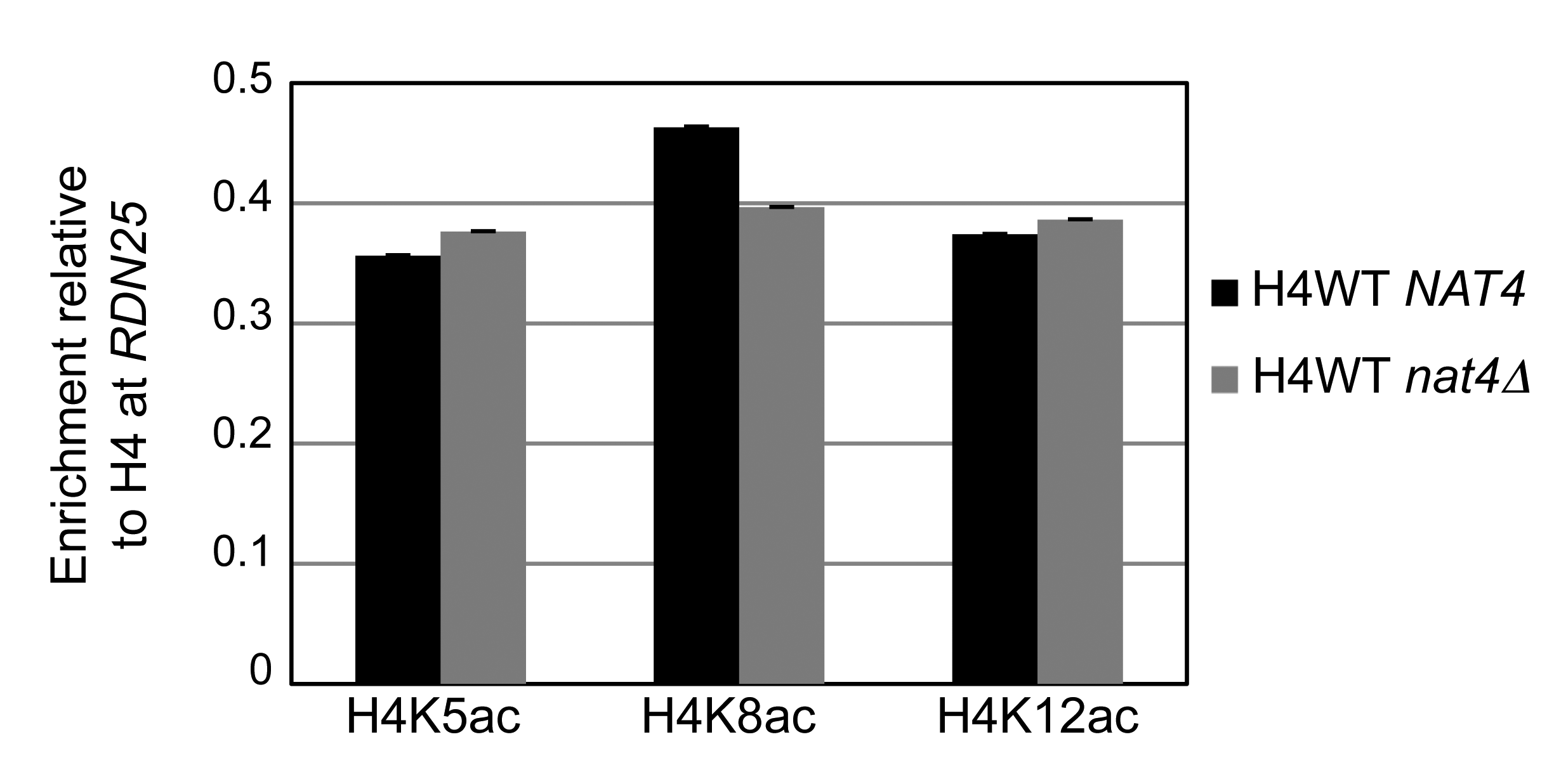

Supplement: Figure S10 — Deletion of NAT4 does not affect the levels of H4K5, 8 or 12 acetylation. ChIP experiments were performed in the indicated strains using antibodies against H4K5ac, H4K8ac and H4K12ac. The immunoprecipitated chromatin was analyzed by quantitative RT-PCR using primers specific to the RDN25 gene. The enrichment from each antibody was normalized to the occupancy of H4. Errors bars indicate s.e.m for duplicate experiments. (TIF) [file pgen.1003805.s010.tif]
