# Supplementary material for: N-alpha-terminal Acetylation of Histone H4 Regulates Arginine Methylation and Ribosomal DNA Silencing
Source: PLoS Genet. 2013 Sep 19;9(9):e1003805. doi: 10.1371/journal.pgen.1003805 (PMC3778019; doi:10.1371/journal.pgen.1003805)
Supplement: Protocol S1 — Additional materials and methods used to construct yeast strains generate antibodies and purify Hmt1. (DOCX) [file pgen.1003805.s011.docx]

**Protocol S1 (Supplementary materials and methods)**

**Plasmid transformation and construction of yeast strains:**

Point mutations in H4 (pMR206) and H2A (pJD150) were generated in plasmids pMR206 (1) and pJD150 (2), respectively, by PCR mutagenesis as previously described (1). *NAT4* catalytic mutants were generated as follows: the entire *NAT4* ORF and the first 200bp of its 3'-UTR were cloned into pCR2.1 TOPO plasmid (Invitrogen), and a C-terminal HA-tag was introduced by PCR on this template. HA-tagged *NAT4* was then used as template to introduce point mutations by PCR in motif A (*nat4cmA-HA*), motif B (*nat4cmB-HA*) or in both (*nat4cmAB-HA*). To integrate the *NAT4-HA* wild-type or point-mutant versions to the genome, a PCR fragment containing 40bp of the *NAT4* 5'-UTR, *NAT4-HA* ORF, 200 bp of the *NAT4* 3'-UTR and the first 40 nucleotides of the KanMX cassette, was transformed in the wild-type JHY6 yeast strain together with another PCR fragment containing the full KanMX4 cassette and 40 bp of the *NAT4* 3'-UTR. The mutants were selected on YPAD+G418 plates and confirmed by sequencing. The following mutations were generated: arginine 194 to histidine, glycine 199 to valine, asparagine 233 to serine and tyrosine 240 to tryptophan. The Y7092 yeast strain was transformed with the MORF-HMT1 plasmid (3) to obtain the strain AK267 that was used for overexpression of the Hmt1-6His-Ha-ZZ protein. H4 mutant strains were constructed as follows: the pMR206 (TRP1-HHT2-HHF2) plasmid bearing the appropriate point mutations on histone H4 (HHF2) was used to transform the *NAT4* and *nat4∆* JHY6 strains. Initial selection was performed on SC-Trp plates, so that the transformed cells can keep the pMS333 (URA3-HHT2-HHF2) plasmid encoding wild-type H4 in order to minimize the appearance of suppressing mutations. Then, the pMS333 plasmid was shuffled out by counterselection on SC-Trp plates containing 5-fluoroorotic acid. The counterselection step was repeated twice and the final strains were tested on YPAD, SC-Ura, SC-Trp and SC+FOA. Using a similar methodology, we constructed the strains AK224 and AK226 from the parental strain UCC1188. All strains used in this study are listed in Table S1.

**Generation of H4R3me2a and N-acH4 antibodies:**

Rabbit polyclonal antibodies were raised in collaboration with Eurogentec (Belgium). For the generation of H4R3me2a the animal was immunized with the keyhole limpet hemocyanin (KLH) conjugated synthetic peptide H2N-SGR (AsymDimethyl) GKG GKG LGK C-CONH2. Serum was affinity-purified by the manufacturer in a two-step procedure. Antibodies specific to the modification were captured on the AF-Amino TOYOPEARL 650 M matrix together with the immunization peptide. After elution with 100 mM glycine (pH 2.5), the antibodies recognizing the peptide in the absence of modification were eliminated with a matrix coupled with the unmodified peptide H2N- SGR GKG GKG LGK C- CONH2. After the purification, antibody specificity was determined by enzyme-linked immunosorbent assay (ELISA) using the specific and non-specific peptides as antigens. The purity of the H4R3me2a antibody was >89% as determined by high pressure-liquid chromatography. The procedure described above was repeated for the generation of the N-acH4 antibody using the KLH-conjugated peptide AcNH-SGR GKG GKG LGK GGA C-CONH2 and the unmodified peptide H2N-SGR GKG GKG LGK GGA C-CONH2. Specificity of the antibody was tested by ELISA and the purity of the N-acH4 antibody was >99.4%.

**Purification of Hmt1-6His-HA-ZZ protein from yeast:**

The AK267 strain was incubated overnight in SC-Ura + 2% Glucose at 30ºC, and then diluted to O.D ~0.2 in 1L SC-Ura containing 2% Ethanol and 3% Glycerol. At OD ~0.8, Galactose was added (2% final) and the culture was grown for 4.5 hours. Cells were first collected and washed with 10 ml of cold lysis buffer (25 mM Tris-HCl pH 8, 300 mM NaCl, 5% Glycerol, 1mM EDTA pH8 containing complete protease inhibitors obtained from Roche), and then resuspended in lysis buffer (1ml/6gr of pellet). Cells were frozen in liquid nitrogen and then were ground with dry ice powder in a coffee bean grinder. The sample was stored at -20ºC overnight for the dry ice to sublime. All the following steps were performed at 4ºC. The proteins in the yeast powder were solubilized by incubating for 15min in 1ml Binding Buffer A (20 mM Tris-HCl pH8, 600mM NaCl, 5% Glycerol, 10 mM Imidazol and protease inhibitors), and then centrifuged at 14000 rpm for 5 minutes. The supernatant was kept, and the lysing procedure was repeated with the remaining pellet using 0.5ml of Binding Buffer. The supernatants were pooled and diluted with an equal volume of Binding buffer B (20 mM Tris-HCl pH8, 5% Glycerol, 10 mM Imidazol and protease inhibitors) before addition of 200ml Nickel-NTA beads (Qiagen). The samples were incubated at 4ºC for 2h. The beads were recovered by centrifugation at 2000 rpm for 2 minutes and washed 2x3min in 2ml Binding Buffer C (20 mM Tris-HCl pH 8, 300mM NaCl, 5% Glycerol, 10-20 mM Imidazol and protease inhibitors). The Hmt1-6His-HA-ZZ protein was eluted in 50-100 µl fresh Elution Buffer (20 mM Tris-HCl pH 8, 50mM NaCl, 5% Glycerol, 250 mM Imidazol and protease inhibitors) with vigorous shaking at 25ºC for 5min. The eluate was separated from the beads by centrifugation and was stored at -20ºC until further analysis by SDS-PAGE, western blotting and *in vitro* methyltransferase assays.

**Supplementary References**

1. Kirmizis A*, et al.* (2007) Arginine methylation at histone H3R2 controls deposition of H3K4 trimethylation. *Nature* 449(7164):928-932.

2. Harvey AC, Jackson SP, Downs JA (2005) Saccharomyces cerevisiae histone H2A Ser122 facilitates DNA repair. *Genetics* 170(2):543-553.

3. Gelperin DM*, et al.* (2005) Biochemical and genetic analysis of the yeast proteome with a movable ORF collection. *Genes Dev* 19(23):2816-2826.
