# Supplementary material for: N-alpha-terminal Acetylation of Histone H4 Regulates Arginine Methylation and Ribosomal DNA Silencing
Source: PLoS Genet. 2013 Sep 19;9(9):e1003805. doi: 10.1371/journal.pgen.1003805 (PMC3778019; doi:10.1371/journal.pgen.1003805)
Supplement: Table S1 — List of yeast strains used in this study. (DOCX) [file pgen.1003805.s012.docx]

Table S1: List of yeast strains

| **Name** | **Genotype** | **Reference** |
| --- | --- | --- |
| BY4741 | *MAT****a****, ura3Δ0, leu2Δ0, his3Δ1, met15Δ0* | Euroscarf |
| AK312 | Same as BY4741, with *set5::KanMX4, nat4::NatMX4* | This work |
| JHY6 | *MAT****a***, *ura3-52*, *lys2-801*, *ade2-101*, *trp*1-289, *his3*Δ*1*, *leu2*-3,112, *Δhhf2-hht2*, Δ*hhf1-hht1*, pMS333*[URA3-HHT2-HHF2]* | Kirmizis *et al*. (2007) Nature 449:928-932 |
| AK236 | Same as JHY6, except *nat4::KanMX4* | This work |
| AK244 | Same as JHY6, except *nat4::NAT4-HA-KanMX4* | This work |
| AK245 | Same as JHY6, except *nat4::nat4cmA-HA-KanMX4* | This work |
| AK246 | Same as JHY6, except *nat4::nat4cmB-HA-KanMX4* | This work |
| AK247 | Same as JHY6, except *nat4::nat4cmAB-HA-KanMX4* | This work |
| AK237 | *MAT****a****, ura3-52, lys2-801, ade2-101, trp1-289, his3Δ1, leu2-3,112, Δhhf2-hht2, Δhhf1-hht1*, pMR206[*TRP1-HHT2-HHF2*] | Kirmizis *et al*. (2007) Nature 449:928-932 |
| AK222 | Same as AK237, except pAK25[pMR206 *HHF2 S1A*] | This work |
| AK238 | Same as AK237, except *nat4::KanMX4* | This work |
| AK239 | Same as AK237, except pAK110[pMR206 *HHF2 K5,8,12R*] | This work |
| AK240 | Same as AK237, except pAK110[pMR206 *HHF2 K5,8,12R*], *nat4::KanMX4* | This work |
| AK326 | Same as AK237, except pAK127[pMR206 *HHF2 R3K, K5,8,12R*]*, nat4::KanMX4* | This work |
| AK327 | Same as AK237, except pAK128[pMR206 *HHF2 S1A, K5,8,12R*] | This work |
| FY406 | *MAT****a,*** *(hta1-htb1)Δ::LEU2, (hta2-htb2)Δ::TRP1, ura3-52,1, leu2Δ1, lys2Δ1, lys2-128Δ, his3Δ200, trp1Δ63*, pAB6[*HTA1-HTB1, URA3*] | Harvey *et al*. (2005) Genetics 170:543-553 |
| FHY2 | Same as FY406, except pJD150[*HTA1-HTB1, HIS3*] | Harvey *et al*. (2005) Genetics 170:543-553 |
| AK234 | Same as FHY2, except pAK27[pJD150 *HTA1 S1A*] | This work |
| YSC5106 WT | *MAT****a****, his3Δ200, leu2Δ0, lys2Δ0, trp1Δ63, ura3Δ0, met15Δ0, can1::MFA1pr-HIS3, hht1-hhf1::NatMX4, hht2-hhf2::*[*HHTS-HHFS*]**-URA3* | Open biosystems |
| AK315 | Same as YSC5106, except *nat4::KanMX4* | This work |
| YSC5106 H4R3K | Same as YSC5106, except *hht2-hhf2::*[*HHTS-HHFS R3K*]*-*URA3* | Open biosystems |
| AK318 | Same as H4R3K, except *nat4:NatMX4* | This work |
| Y10000 pBEVY-U | *MAT****α****, his3Δ1; leu2Δ0; lys2Δ0; ura3Δ0* pBEVY-U | Hole *et al*. (2011)  PLoS One 6:e24713 |
| Y16202 pBEVY-U | Same as Y10000 pBEVY-U, except *nat4::kanMX4* | Hole *et al*. (2011)  PLoS One 6:e24713 |
| Y16202 pBEVY-U-h*NAA40* | Same as Y16202 pBEVY-U, except pBEVY-U*-*h*NAA40* | Hole *et al*. (2011)  PLoS One 6:e24713 |
| UCC1188 | *MAT****α,*** *leu2Δ1, lys2-801, trp1, ura3, hhf1-hht1::LEU2 hhf2-hht2::HIS3 RDN1::URA3,* pMP9[*LYS2 CEN ARS*]*-HHF2-HHT2* | Van Leeuwen *et al*. (2002) Cell 109:745-756 |
| AK208 | Same as UCC1188, except *nat4::NatMX4* | This work |
| AK224 | Same as UCC1188, except pMR206[*TRP1-HHT2-HHF2*] | This work |
| AK226 | Same as UCC1188, except pAK25[pMR206 *HHF2 S1A*] | This work |
| UCC1369 | *MAT****a,*** *ade2Δ::hisG, his3Δ200, leu2Δ0, lys2Δ0, met15Δ0, trp1Δ63, ura3Δ0, adh4::URA3-TEL(VII-L), ADE2-TEL(V-R), Δhhf2-hht2::MET15, Δhhf1-hht1::LEU2,* pMP9[*LYS2 CEN ARS*]*-HHF2-HHT2* | Van Leeuwen *et al*. (2002) Cell 109:745-756 |
| AK210 | Same as UCC1369, except *nat4::NatMX4* | This work |
| UCC7262 | *MAT****a,*** *ade2 his3 leu2 lys2 ura3 ADE2-TEL(V-R) hmra::URA3, hhf2-hht2::MET15, hhf1-hht1::LEU2,* pMP9[*LYS2 CEN ARS*]*-HHF2-HHT2* | Van Leeuwen *et al*. (2002) Cell 109:745-756 |
| AK207 | Same as UCC7262, except *nat4::NatMX4* | This work |
| UCC7266 | *MAT****a,*** *ade2 his3 leu2 lys2 ura3 ADE2-TEL(V-R) hmlα::URA3, hhf2-hht2::MET15, hhf1-hht1::LEU2,* pMP9[*LYS2 CEN ARS*]*-HHF2-HHT2* | Van Leeuwen *et al*. (2002) Cell 109:745-756 |
| AK209 | Same as UCC7266, except *nat4::NatMX4* | This work |
| AK267 | *MAT****α****, can1Δ::STE2pr-Sp-his5, lyp1Δ, his3Δ1, leu2Δ0, ura3Δ0, met15Δ0,* pMORF[*HMT1-6xHis-HA-ZZ*] | This work |
| AK410 | Same as AK237, except pAK130[pMR206 *HHF2 S1P*] | This work |
