# Supplementary material for: N-alpha-terminal Acetylation of Histone H4 Regulates Arginine Methylation and Ribosomal DNA Silencing
Source: PLoS Genet. 2013 Sep 19;9(9):e1003805. doi: 10.1371/journal.pgen.1003805 (PMC3778019; doi:10.1371/journal.pgen.1003805)
Supplement: Table S2 — List of primer sequences used for qRT-PCR. (DOCX) [file pgen.1003805.s013.docx]

Table S2: List of primers

| **Primers** | **Forward (5'- 3')** | **Reverse (5'- 3')** |
| --- | --- | --- |
| A | GGGTAACCCAGTTCCTCACTA | GCATATATTTCTTGTGTGAGAAAGG |
| B | CCGTTATTGGTAGGAGTGTGG | TAACATCCCAATGCGGACTA |
| C *(RDN5)* | TGGTAAGAGCCTGACCGAGT | GATTGCAGCACCTGAGTTTC |
| D | TACACCCTCGTTTAGTTGCTTCT | CGGTATGCGGAGTTGTAAGA |
| E | AGAACGCGGTGATTTCTTTG | GGACGCCTTATTCGTATCCA |
| F | GGTGATTTCTTTGCTCCACA | TGCTAGCCTGCTATGGTTCA |
| G | GACCCGAAAGATGGTGAACT | CCAGAGTTTCCTCTGGCTTC |
| H | TGCGAGTGTTTGGGTGTAAA | ATCCGAAGACATCAGGATCG |
| I *(RDN25)* | TTGACTTACGTCGCAGTCCTCAGT | AGGACGTCATAGAGGGTGAGAATC |
| *J (RDN58)* | AACGGATCTCTTGGTTCTCG | TGTGCGTTCAAAGATTCGAT |
| K | CTGGGCAAGAAGACAAGAGA | CACCGTTTGGAATAGCAAGA |
| L *(RDN18)* | TCAGAGCGGAGAATTTGGAC | GGTTCACCTACGGAAACCTT |
| M | CTGGTTGATCCTGCCAGTAG | TAATGAGCCATTCGCAGTTT |
| N *(RDN37)* | CAAGAGGGAATAGGTGGGAAAA | GACAAGCATATGACTACTGGCA |
| *RPP0* | AACGGTCAAGTGTTCCCATC | AGCGGAAACGAAGTGAGAAA |
